# Phage tail protein methylation enabling the virus to bind to the surface of bacterial cells is vital for progeny infectivity

**DOI:** 10.1016/j.cellin.2026.100345

**Published:** 2026-07-01

**Authors:** Sijia Fan, Junlong Li, Yuyun Wei, Yuxuan Zhang, Xiao Guo, Di Wu, Zheng-Guo He

**Affiliations:** a College of Life Science and Technology, Guangxi University, Nanning, Guangxi, 530004, China; b State Key Laboratory of Virology and BioSafety, Taikang Center for Life and Medical Sciences, TaiKang Medical School (School of Basic Medical Sciences), Wuhan University, Wuhan, Hubei, 430071, China; c State Key Laboratory of Metabolism and Regulation in Complex Organisms, TaiKang Center for Life and Medical Sciences, School of Basic Medical Sciences, Wuhan University, Wuhan, Hubei, 430071, China

**Keywords:** Phage, Post-translational modification, Methyltransferase, Antiphage defense, Phage adsorption

## Abstract

The importance of methylation-based protein modifications in viral infection remains largely unknown. Here, we show that phage tail protein methylation depending on a host-encoded enzyme, MepM, is vital for maintaining the viral infectivity. Inactivation of the *mepM* gene of *Mycolicibacterium smegmatis* (*M*. *smegmatis*) confers broad-spectrum phage resistance to the host. The absence of *mepM* does not affect primary infection but abolishes progeny phage infectivity. The phage tail protein Gp34 expressed in wild-type strain, but not in *mepM-*deleted strain, undergoes unique MepM-dependent methylation-based modifications at its ELS amino acid motif, enabling it to maintain strong binding to the surface of mycobacterial cells. Recombinant phages harboring mutant genes encoding Gp34 with altered amino acid residues at its methylation sites do not exhibit infectivity. Our findings provide original experimental data on the previously undefined mechanism by which bacteriophages utilize host-encoded enzymes for protein methylation to maintain infectivity.

## Highlights

• Phage tail protein Gp34 expressed in the *mepM*-deleted strain exhibit adsorption defects.
• The tail proteins undergo methylation modification enabling the virus to maintain strong binding to the bacterial surface.
• MepM-dependent methylation of tail proteins is essential for the infectivity of progeny phages.

## Graphical abstract

**Figure:**
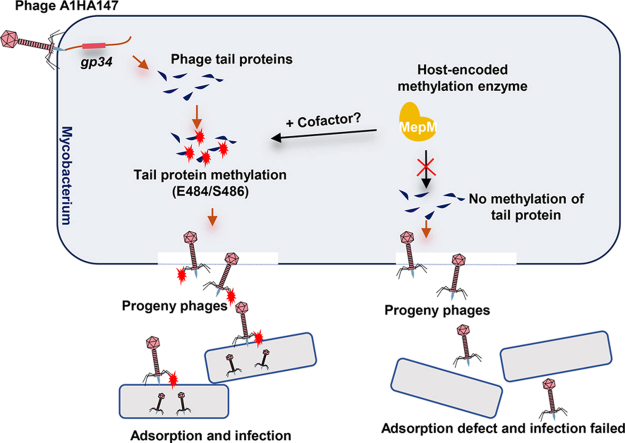

## 1 Introduction

As obligate intracellular parasites, viruses rely entirely on host cells for their replication and the assembly of new virions (Salmond & Fineran, 2015). Bacteriophages or phages, which consist primarily of a protein coat and an enclosed nucleic acid genome (DNA or RNA), are viruses that specifically infect bacteria (Dion et al., 2020). During bacterial cell infection, the bacteriophage first recognizes specific receptors on the bacterial surface via its tail fibers (or spikes) and subsequently injects its nucleic acid genome into the host cytoplasm (Nobrega et al., 2018). Subsequently, the phage genome replicates and viral capsid proteins are synthesized within the host cell, followed by assembly of progeny phages and their release via either host-cell lysis or extrusion (Penadés et al., 2025). In the long-term battle for survival, hosts have developed various mechanisms to counteract phage infection, including the prevention of viral adsorption or DNA injection, interference with phage gene transcription and DNA replication, blockage of progeny phage release, and the induction of abortive infections (Hampton et al., 2020). In response to these host defenses, phages have also developed several evasion systems, such as the anti-RM and anti-CRISPR systems (Georjon & Bernheim, 2023; Davidson, 2017; Mahler et al., 2025).

Viral protein post-translational modifications (PTMs) represent a crucial strategy for viral adaptation to the host environment, as well as for the regulation of their life cycle and host defense counteraction (Chamontin et al., 2021; Chen et al., 2022; Kumar et al., 2020; Loboda et al., 2019). When eukaryotic viral proteins undergo host-mediated modifications, they help the virus localize to appropriate cellular compartments, thereby enhancing packaging and release efficiency (Cheng et al., 2022; Liu & Yang, 2025). For instance, the SARS-CoV-2 spike S protein undergoes glycosylation upon binding to the host ACE2 receptor, and this results in glycan coverage across the viral surface (Zhao et al., 2020). This facilitates viral entry into cells via membrane fusion or endocytosis, ultimately leading to the evasion of attack by the immune system. Of note, host-mediated modification of viral structural or regulatory proteins can effectively induce the initiation of viral transcription, translation, or packaging within the host. For example, phosphorylation of the SARS-CoV-2 nucleo-capsid protein enhances its selective binding to viral RNA, promoting viral replication and transcription, thereby increasing viral load in the host (Cheng et al., 2022). Additionally, modified eukaryotic viral proteins can conceal antigenicity, interfere with host signaling pathways, evade degradation by the host, and avoid recognition by restriction factors. For instance, the dengue virus has developed multiple strategies to manipulate the innate immune response of the host (St. John & Rathore, 2019).

Bacteria can utilize PTMs as a defense strategy against phage infection. For instance, the *Pseudomonas aeruginosa* glycosyltransferase, TfpO, masks the phage receptor pilin protein, PilA, through glycosylation, preventing phage adsorption to type IV pilin (Harvey et al., 2017). Phosphorylation of the *Staphylococcus aureus* protein, Stk2, which is activated by the phage phiNM1 protein, PacK, induces abortive infection, thereby blocking phage infection (Depardieu et al., 2016). Recent studies have shown that bacteria can manipulate phage assembly via PTMs. For example, host bacteria covalently link the ubiquitin-like protein, Bil, to the phage central tail fiber proteins, SECphi27 and SECphi4, thereby inducing ubiquitin-like modifications which result in the development of tailless phages and phages with Bil-coupled central tail fibers, neither of which can complete final assembly (Hör et al., 2024). PfkA/PfkB phosphorylation in the *P. aeruginosa* Pf6 prophage drives the lysogenic-lytic switch of Pf4 and Pf6 prophages (Guo et al., 2024). Conversely, PTMs have been reported to aid phage replication cycle completion and host defense evasion. For example, the acetyltransferase, AcrVA5, encoded by the *Moraxella bovoculi* prophage, acetylates the Cas12a protein, enabling CRISPR-Cas12 system evasion (Dong et al., 2019). The mycobacteriophage, Che8, encodes a glycosyltransferase within the host cell; upon linkage to oxygen, it glycosylates the phage capsid protein, allowing normal host infection and protecting Che8 from antibody recognition (Freeman et al., 2023).

Methyltransferases can transfer a methyl group (-CH3) from a donor compound to a recipient compound, and these modifications have mainly been reported for phage or host nucleic acids. In the host bacterial restriction modification (R-M) system, the host methylates its own DNA whilst cleaving and degrading exogenous unmethylated phage DNA to prevent phage infection (Gao et al., 2020). For example, *Marinomonas mediterranea* MMB-2 uses the Type I RM defense system (Mme2I) to degrade unmethylated exogenous DNA and prevent the assembly of several new phages in the *Murciavirus* genus (Martinez-Cazorla et al., 2025). Bacteriophages also utilize host methyltransferases to methylate phage DNA, thereby preventing recognition cleavage by restriction endonucleases (Went et al., 2024). For example, bacteriophage KHP30T receives a methyl group through the action of host-encoded methyltransferases during multiple generations of infection, and this enhances its infection efficiency against the host bacteria (Takahashi et al., 2025). At present, only sporadic reports on host methylation and eukaryotic viral protein PTMs are available. As an example, the SARS-CoV-2 nucleocapsid protein can be methylated to promote its binding to viral RNA and facilitate viral particle production (Zheng et al., 2023). However, there have been no reports on phage protein methylation by a host enzyme, and it is unclear whether this PTM exists; in addition, it is unclear how this modification affects phage infection.

In this study, an *MSMEG_6482*-encoded protein essential for phage infection, designated MepM, was identified in *M*. *smegmatis*. Disruption of *mepM* confers broad-spectrum antiphage ability to the mycobacterium. Interestingly, *mepM* deletion does not affect primary infection of phages but abolishes progeny phage infectivity. Further, we found that the phage tail protein undergoes MepM-dependent methylation in mycobacteria and that PTMs are essential for viral infectivity. Therefore, this study identified methylation-based modification of phage proteins as the previously undefined mechanism of viral infectivity maintenance, significantly improving our understanding of the mechanisms underlying virus-host interactions.

## 2 Results

### 2.1 *mepM* disruption confers broad-spectrum phage resistance to the host

**Fig. 1:**
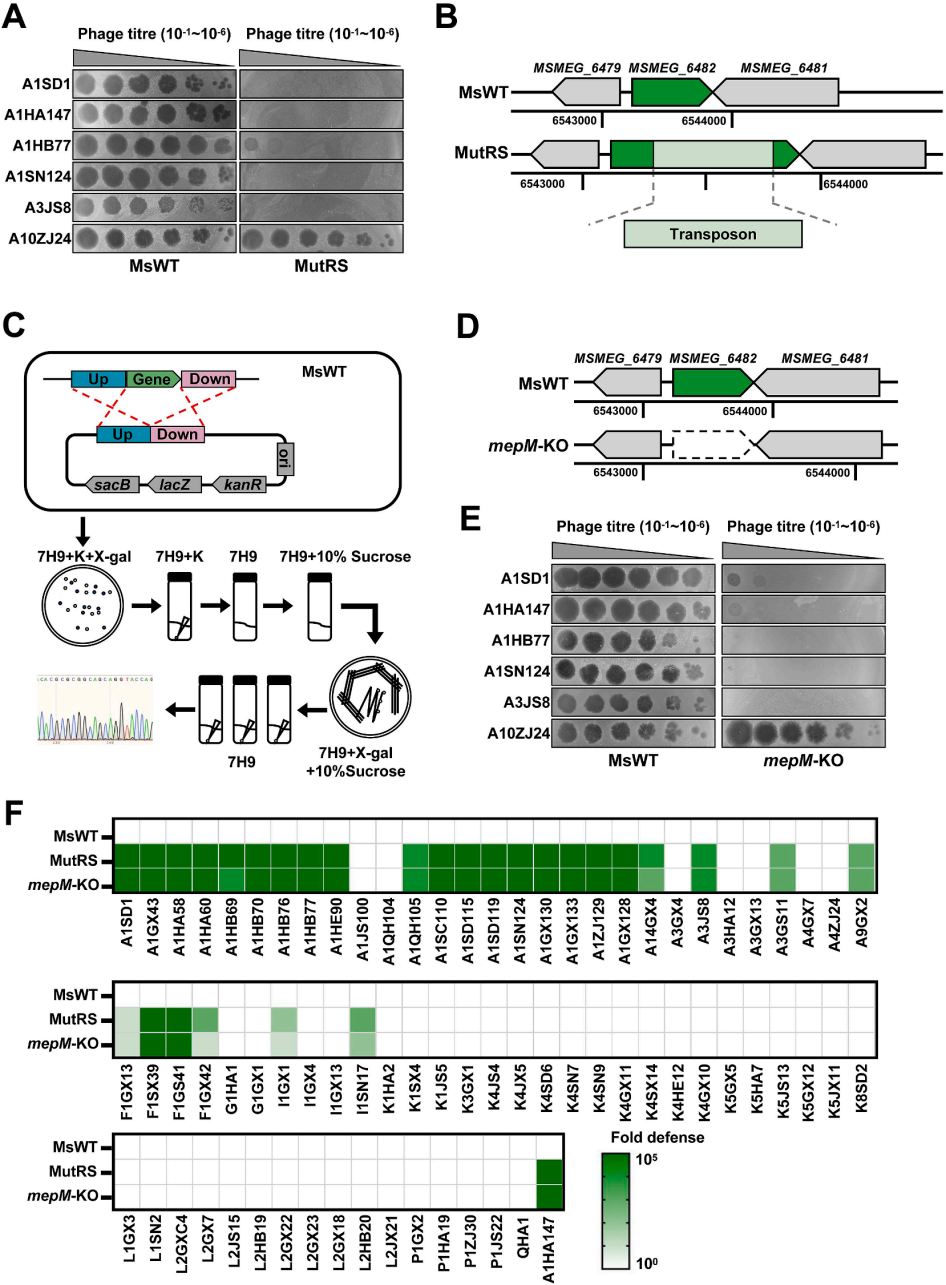
***mepM*****disruption confers the host broad-spectrum phage resistance.** **(A)** Analysis of phage resistance in the MutRS strain. The phages were diluted in a 10-fold gradient to obtain a range of 10^−1^ to 10^−6^ and spotted on double-layered plates containing wild-type and mutant strains of *M*. *smegmatis* MutRS, and cultured for 24 h **(B)** Schematic of the MutRS mutation site in the mutant *M. smegmatis* strain. The genome of the mutant strain harbors a transposon insertion within *mepM* (also see Fig. S1A). **(C)** Principle and process diagram for the traceless knockout strategy. The upstream and downstream segments of the target gene were cloned into the pMind-*lacZ-sacB* vector, electroporated into the wild-type *M*. *smegmatis* (MsWT), and cultured on plates containing Kanamycin and X-gal. Blue colonies were first selected and cultured under sucrose pressure. White colonies were subsequently selected for validation. **(D)** Schematic of the ORF structure of the knockout strain, *mepM*-KO. **(E)** Assays for the antiphage activity of the knockout strain, *mepM*-KO. The phages were diluted in a 10-fold gradient to obtain a range of 10^−1^ to 10^−6^ and spotted on double-layered plates containing the WT and *mepM*-KO strains. *mepM*-KO resistance to phages is similar to that of the MutRS strain. **(F)** Antiphage heatmap for the *mepM*-KO and MutRS strains. The growth of various mycobacteriophages on double-layered plates containing three different mycobacterial strains was measured separately (Fig. S1E). Different shades of green in the Heatmap represent the antiphage abilities of the strains, with darker shades representing stronger resistance.

Our discovery stemmed from the isolation of a mutant *M. smegmatis* strain (MutRS), which exhibited broad-spectrum resistance to phage infection. As shown in Fig. 1(A), when various mycobacteriophages were serially diluted tenfold and spotted onto double-layered plates containing wild-type *M. smegmatis* (MsWT), clear plaques were formed on all plates. Conversely, the mycobacteriophages failed to produce observable plaques on plates containing the MutRS strain under similar experimental conditions. Moreover, the majority of phages exhibited no detectable lytic activity even at their highest concentration (10^−1^) (Fig. 1(A)), suggesting that the MutRS strain exhibits extensive resistance to mycobacteriophages. Subsequently, we conducted a whole-genome sequencing analysis on the strain to investigate the potential mechanism of this resistance. We identified a transposon insertion at the *mepM* (*MSMEG_6482*) locus in the MutRS genome, and this disrupted the open reading frame (ORF) of the gene (Fig. 1(B)). Furthermore, to determine whether this MutRS resistance was caused by the *mepM* insertion disruption, we carried out a scarless knockout of *mepM* from the *M. smegmatis* genome through homologous recombination (Fig. 1(C)), successfully obtaining the *mepM* knockout strain, *mepM-*KO (Fig. 1(D) and Fig. S1A). The morphology and growth characteristics of the *mepM-*KO strain were not significantly different from those of the MsWT strain (Fig. S1B–D). Upon comparing the sensitivities of the MutRS and *mepM-*KO strains to the aforementioned phages, *mepM-*KO and MutRS were found to exhibit similar phage resistance spectra on double-layered agar plates (Fig. 1(E)). Subsequently, we extensively investigated differences in sensitivity between the three strains—MsWT, MutRS, and *mepM-*KO, to 80 different mycobacteriophages. The resistance spectra for MutRS and *mepM-*KO were highly similar, with both exhibiting significant resistance to the A1, A3, A9, F1, and I1 sub-cluster mycobacteriophages, especially to the A1 and F1 sub-clusters, exhibiting a 10^5^-fold increase in resistance to the MsWT strain (Fig. 1(F) and Fig. S1E).

Collectively, these results demonstrate that insertional inactivation or deletion of *mepM* confers mycobacteria with broad-spectrum resistance to mycobacteriophages.

### 2.2 MepM is a widely conserved protein containing methyltransferase residues essential for phage infection

**Fig. 2:**
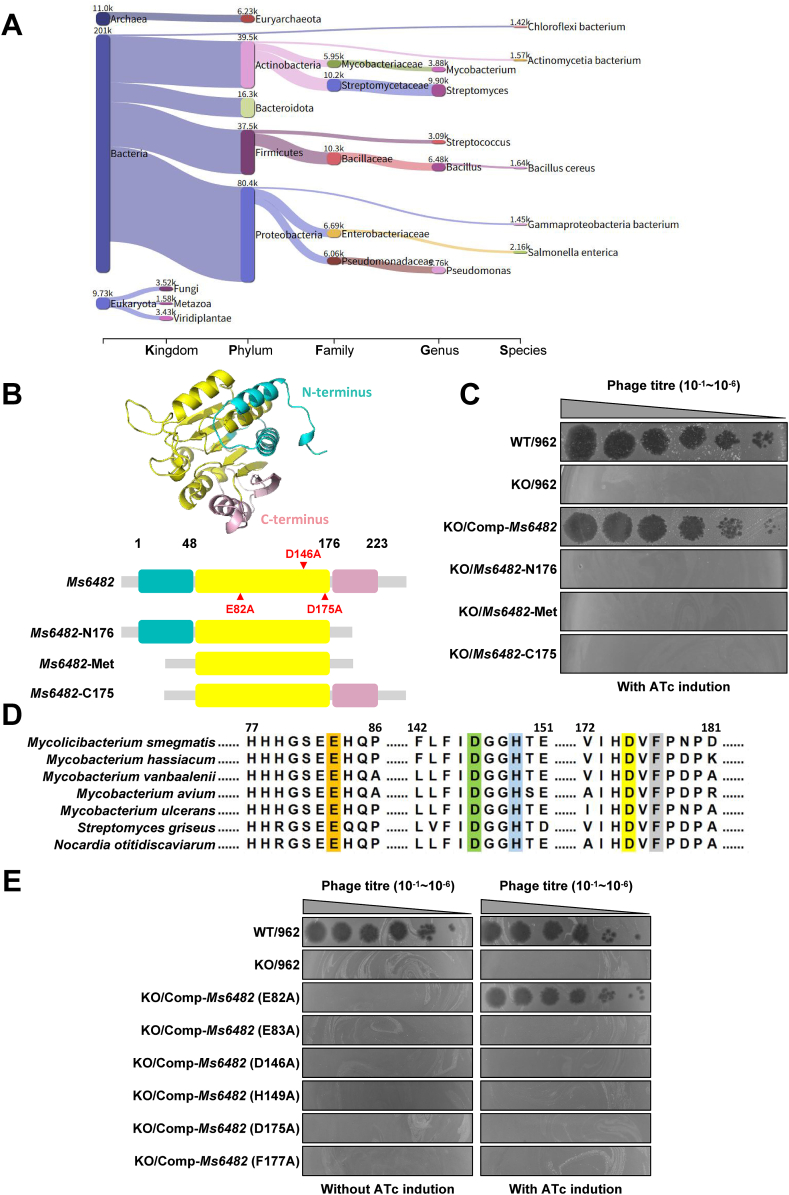
**Conserved methyltransferase MepM residues are essential for phage infection.** **(A)** Visualization of the Last Common Ancestor (LCA) of all AFDB structural homologies of MepM presented as a Sankey plot generated using Pavian. Only the 5 largest taxonomical nodes per rank are displayed. **(B)** Three-dimensional structural simulation and domains of methyltransferase MepM. Using the methyltransferase of *M. hassicaum* as a template, the MepM structure was simulated using AlphaFold3. Its amino acid residues 49∼176, 1∼48, and 177∼223 belong to the methyltransferase domain (yellow part), the N-terminal domain (blue part), and the C-terminal domain (pink part), respectively. **(C)** Restoration effect of truncated gene fragments on the antiphage ability of the *mepM*-KO strain. Several truncated gene fragments were expressed in the *mepM*-KO strain. The A1HA147 phage was diluted in a 10-fold gradient to obtain a range of 10^−1^ to 10^−6^ and each dilution was spotted on double-layered plates containing the KO/Comp-Ms6482, KO/Ms6482-N176, KO/Ms6482-Met, or KO/Ms6482-C175 strains, induced by 200 ng/mL ATc. **(D)** Conservation analysis of the methyltransferases of different bacteria. The conserved amino acids of the protein are highlighted with different colors. **(E)** Restoration effect of the conserved amino acid mutations in *mepM* on the antiphage ability of the *mepM*-KO strain. The mutant gene containing a single conserved amino acid mutation, such as E82A, E83A, D146A, H149A, D175A, or F177A, was complemented into and expressed in the knockout strain. The A1HA147 phage was diluted and spotted on double-layered plates containing the aforementioned recombinant strains for the observation of plaque formation, induced by 200 ng/mL ATc.

Next, we investigated the taxonomic composition of MepM and the conservation of its domain based on structural similarity and Last Common Ancestor (LCA) analyses. Upon mapping the best matches onto the Tree of Life and identifying the respective most recent common ancestors, MepM was found to have structural homologs in all three domains—the archaea, bacteria, and eukaryota domains (Fig. 2(A)). Furthermore, upon further analysis of MepM distribution in viruses, its structural homologs were not only widely found in phages but also in various eukaryotic viruses (Fig. S2A), indicating that it is a widely conserved protein. Through AlphaFold for structural comparison analyses, amino acids 49∼176 of the MepM protein were found to belong to a MetT-like methyltransferase domain (Fig. 2(B), yellow). Subsequently, we induced the expression of three truncated mutants of the gene and determined whether they could reverse the *mepM-*KO strain phage phenotype (Fig. 2(C)). When the 10-fold dilution of the A1HA147 phage was dropped onto a plate containing wild-type *M. smegmatis*, anhydrous tetracycline (ATc)-induced full-length gene fragment expression almost completely restored *mepM-*KO sensitivity to phages; conversely, neither the MepM-N terminus, the MepM-C terminus, nor the MetT domain exhibited this ability, and these recombinant strains still maintained their phage-resistant phenotypes (Fig. 2(C)). Similarly, *mepM-*KO strain resistance to the F-cluster phage, F1SX39, could also be abolished through the expression of the full-length gene fragment; however, none of the three truncated fragments had this effect (Fig. S2B–D). Therefore, the complete *mepM* fragment is necessary for the restoration of the antiphage phenotype of the *mepM-*KO strain.

Furthermore, BLAST analysis revealed that *mepM* has corresponding homologous genes in multiple non-tuberculous mycobacterial species, such as *M. hassiacum*, *M. vanbaalenii*, *M. avium*, and *M. ulcerans*, as well as in *Streptomyces* and *Nocardia*species. Particularly, the multiple amino acid residues of the methyltransferase domain encoded by this gene—E83, D146, H149, D175, and F177—exhibited high conservation (Fig. 2(D)). Next, these mutant genes, which harbored a single residue mutation (Fig. S2E), were expressed (the non-conserved amino acid residue, E82, was used as a control) to determine their effects on the antiphage phenotype of the *mepM-*KO strain. Clearly, point mutations of these conserved amino acids (E83A, D146A, H149A, D175A, and F177A) could not restore the infectious ability of the phages under ATc induction, and could not induce the formation of clear phage lysis spots on the double-layered plate (Fig. 2(E) and Fig. S2D). However, under similar experimental conditions, expression of the gene harboring a point mutation (E82A) at the non-conserved residue E82, as well as the expression of the full-length gene, neutralized the resistance phenotype of the *mepM-*KO strain. Both phages could induce the formation of clear lysis spots on the double-layered plate (Fig. 2(E) and Fig. S2D), suggesting that the presence of conserved amino acid residues in the methyltransferase domain is necessary for the restoration of the *mepM-*KO strain antiphage phenotype.

Therefore, our results demonstrate that MepM is a widely conserved protein and that the expression of full-length *mepM* in the *mepM-*KO strain is necessary for the restoration of phage infectivity in the mutant strain. Mutations of the conserved amino acid residues of the methyltransferase domain led to a loss of the ability of the gene to restore the *mepM-*KO strain phage-resistant phenotype.

### 2.3 *mepM* deletion does not affect the primary phage infection

**Fig. 3:**
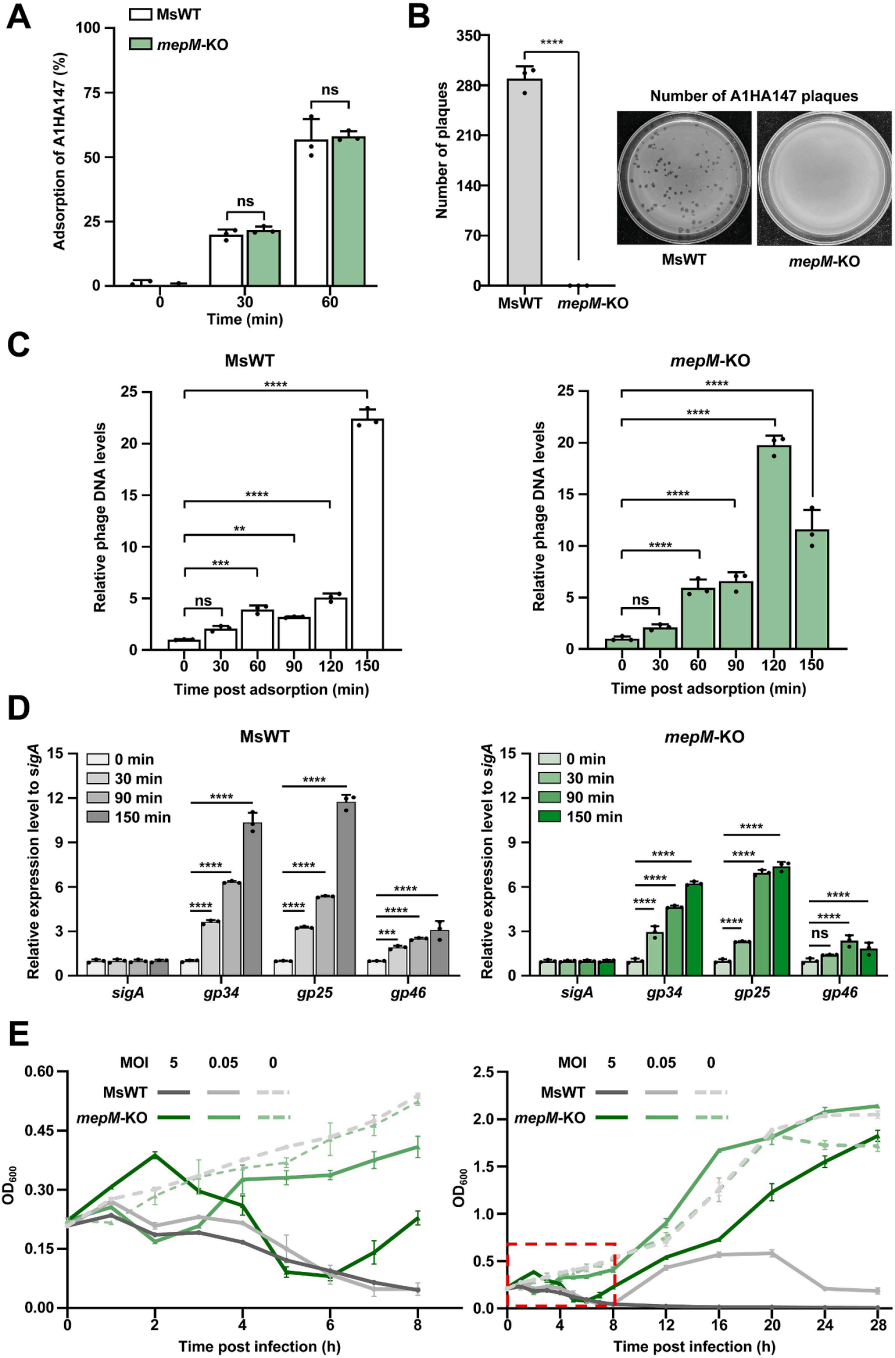
***mepM*****deletion does not affect the primary infection but causes infection defects in progeny phages.** **(A)** Determination of the adsorption capacity of the A1HA147 phage on wild-type and *mepM*-KO strains. Biological replication: *n* = 3. (ns indicates no significant difference) **(B)** Determination of plaque formation ability after electroporation of A1HA147 phage genomic DNA into wild-type and *mepM*-KO strains. Biological replication: *n* = 3. (∗∗∗∗ indicates *p* < 0.000 1). **(C)** Determination of phage DNA abundance in wild-type and *mepM*-KO strains following infection with A1HA147. qPCR was used to quantitatively detect phage DNA copy number in bacterial samples at different time points. Biological replication: *n* = 3. (ns indicates no significant difference, ∗∗ indicates *p* < 0.01, ∗∗∗ indicates *p* < 0.001, ∗∗∗∗ indicates *p* < 0.000 1). **(D)** Determination of phage gene expression in wild-type and *mepM*-KO strains following infection with A1HA147. Gene expression levels for infective phages in bacterial samples were quantitatively detected at different time points through qRT-PCR. *gp46* is an early gene that encodes DNA polymerase I; *gp34* is a late gene that encodes the small tail protein; Gp25 is a tail assembly chaperone protein. *sigA* was used as a control. Biological replication: *n* = 3. (∗∗∗∗ indicates *p* < 0.000 1). **(E)** Growth curves for wild-type and *mepM*-KO strains under infection with the A1HA147 phage. Bacterial counts were determined every hour from 0 to 8 h following infection (left), and every 4 h from 8 to 28 h following infection (right). All data in the quantitative analysis are expressed as mean ± standard deviation. Biological replication: *n* = 3. Two-factor analysis of variance was performed using the GraphPad Prism software for *p*-value calculation. The asterisk indicates a significant difference between the two groups.

We further examined the effects of *mepM* knockout on various stages of phage infection, including adsorption, DNA injection, replication, and gene transcription. First, the adsorption rates of the two phages, A1HA147 and F1SX39, to both the WT and *mepM-*KO strains were measured. As shown in Fig. 3(A), at an MOI of 0.01, the adsorption rate of the A1HA147 phage to the *mepM-*KO strain was 0%, 21.73%, and 58.05% at 0, 30, and 60 min, respectively, with no significant differences observed in adsorption rate between this strain and the WT strain (0%, 19.91%, and 56.83%) at these time points, indicating that the A1HA147 phage could adsorb to the *mepM-*KO strain. Similar results were obtained following infection with the F1SX39 phage (Fig. S3A). Next, to determine whether the *mepM-*KO strain could regain sensitivity to the phage, phage genomic DNA was directly transformed into that of the *mepM-*KO strain through electroporation. As shown in Fig. 3(B) and Fig. S3B, 200∼300 plaques were formed on double-layered plates containing the WT strain following phage DNA introduction; however, no plaques were observed on plates containing the *mepM-*KO strain. This indicates that the *mepM* deletion-induced resistant phenotype does not result from phage adsorption or injection defects.

Quantitative Real-Time PCR (qPCR) was subsequently used to determine whether *mepM* knockout affected the phage replication process. As shown in Fig. 3(C), within one phage infection cycle (90∼150 min), phage DNA abundance at 150 min following infection reached 22.44-fold in the MsWT strain (Fig. 3(C), left), while relative phage DNA abundance reached 19.78-fold in the *mepM-*KO strain at 120 min following infection (Fig. 3(C), right), and this was slightly lower than that at the peak replication time in the WT strain. Similar results were also observed for the F1SX39 phage (Fig. S3C). Therefore, both the A1HA147 and F1SX39 phages could undergo normal, complete DNA replication in the *mepM-*KO strain.

Furthermore, to determine whether there were any abnormalities in the expression of phage genes in *mepM-*KO strains, Quantitative Reverse Transcription PCR (RT-qPCR) was used to monitor the expression levels of representative early genes such as the DNA polymerase I-encoding gene, *gp46*, late genes such as the minor tail protein-encoding gene, *gp34*, and the tail assembly chaperone-encoding gene, *gp25*, following phage infection. As shown in Fig. 3(D), *gp34* expression levels for the A1HA147 phage increased up to 10-fold in the MsWT strain 150 min following infection, while *gp25* and *gp46* expression increased 11.7 and 3.0-folds, respectively (Fig. 3(D), left). Although overall phage gene expression levels in the *mepM-*KO strain were slightly lower than those in the MsWT strain, their levels significantly increased with respect to those observed at the early stage of infection (Fig. 3(D), right), indicating that phage genes exhibited normal transcriptional expression both in MsWT and *mepM-*KO strains. Similar results were obtained for the F1SX39 phage (Fig. S3D).

Therefore, these results indicate that *mepM* deletion does not affect primary infection and phage replication in *mepM-*KO strains.

### 2.4 Progeny phages lose their ability to lyse the *mepM*-KO strain

Next, to determine whether *mepM-*KO strain is sensitive to phages during continuous infection, we monitored the growth rate of host strains under different MOI conditions. As shown in Fig. 3(E), the WT strain multiplied normally prior to phage infection (gray dashed line); however, following infection with A1HA147, irrespective of whether MOI was high (MOI = 5; black solid line) or low (MOI = 0.05; gray solid line), clear growth inhibition of the MsWT strain was observed, with the OD_600_ maintained at 0, indicating that phages could continuously infect and lyse the MsWT strain. The *mepM-*KO strain exhibited normal growth prior to phage infection (green dashed line). However, following infection with the A1HA147 phage at a high MOI of 5 (solid dark green line) until the 6th hour, the OD_600_ for the *mepM-*KO strain first exhibited an increasing trend and then decreased, while the OD_600_ for the *mepM-*KO strain following infection with phages at a low MOI of 0.05 (solid fluorescent green line) first exhibited a decreasing trend and then increased. These results indicated that the phages could complete the first round of infection to produce and release progeny phages (Fig. 3(E), left). After 6 h, there was no inhibition of the knockout strain under both high and low MOI conditions, indicating that the progeny phages lost their ability to continuously infect and lyse mycobacteria. Similar results were obtained following infection with the F1SX39 phage (Fig. S3E).

These results indicated that phages could complete the first round of infection in *mepM-*deficient strains and produce progeny phages; however, these progeny phages lost the ability to lyse the *mepM-*KO strains in subsequent rounds of infection.

### 2.5 Tail protein mutation restores phage infectivity towards the *mepM*-KO strain

**Fig. 4:**
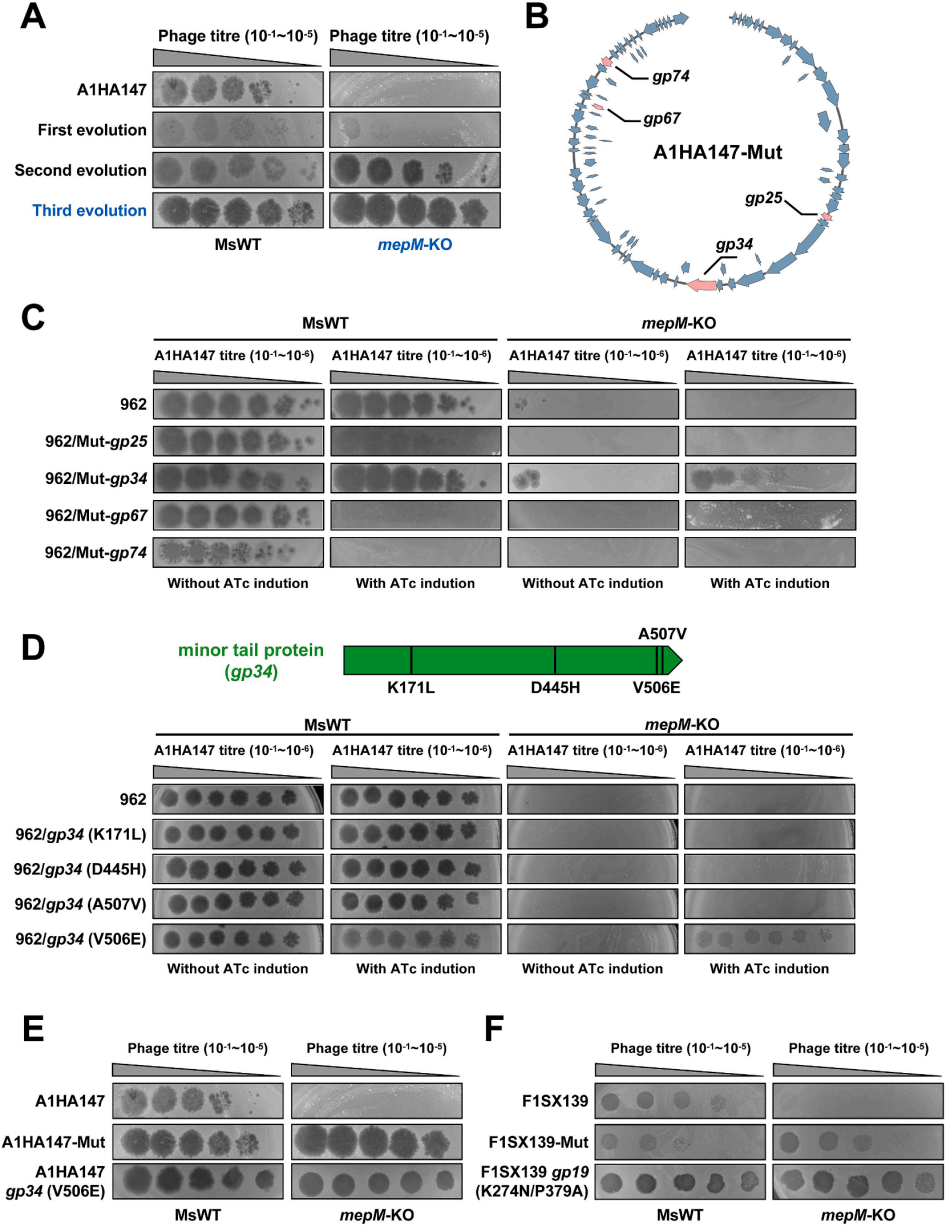
**Tail protein mutation restores phage infectivity toward the*****mepM*****-KO strain.** **(A)** A1HA147 phage evolution causes restoration of infectivity toward the *mepM*-KO strain. Serial phage dilutions (10^−1^∼10^−5^) after three rounds of evolution were spotted on double-layered plates containing wild-type and *mepM*-KO strains. The third generation of evolutionary phages could produce clear lytic plaques. **(B)** Schematic showing the mutated gene in the evolutionary A1HA147-Mut phage. Four mutated genes within the A1HA147-Mut gene (*gp34*, *gp25*, *gp74*, and *gp67*) are marked in pink. **(C)** Mut-*gp34* expression in the *mepM*-KO strain could partially restore A1HA147 phage infectivity. Several mutant genes, including Mut-*gp34*, *gp25*, *gp74*, and *gp67*, were expressed in wild-type and *mepM*-KO strains. Serial phage dilutions (10^−1^∼10^−5^) were spotted on double-layered plates containing different bacterial strains for the determination of infectivity, induced by 200 ng/mL ATc. **(D)** Gp34-V506E mutant gene expression confers the A1HA147 phage with infectivity towards the *mepM*-KO strain. Several mutant genes containing single-residue mutations, such as K171L, D445H, V506E, and A507V, were expressed in wild-type and *mepM*-KO strains. Serial phage dilutions (10^−1^∼10^−5^) were spotted on double-layered plates for the determination of infectivity, induced by 200 ng/mL ATc. **(E-F)** Infection efficiencies of the recombinant phages, A1HA147-*gp34* (V506E) (E) and F1SX39-*gp19* (K274N/P379A) (F), in the *mepM*-KO strain. Wild-type and recombinant A1HA147-*gp34* (V506E) or F1SX39-*gp19* (K274N/P379A) phages were diluted and spotted on double-layered plates for the determination of infectivity.

Next, continuous phage evolution experiments were conducted to identify potential viral genes that cause functional impairment of progeny phage infectivity. As shown in Fig. 4(A), the mutant phage, A1HA147-Mut, was successfully isolated after three rounds of evolution and regained the ability to infect the knockout strain, with a 10^5^-fold increase observed in its infection ability in comparison to that of the WT A1HA147 phage. Genome sequencing revealed mutations in four genes in the A1HA147-Mut genome, i.e., *gp34, gp25, gp74*, and *gp67* (Fig. 4(B)). Among these genes, *gp34* encodes a small tail protein that has a mutant harboring mutations in four amino acid residues. Using a similar evolution strategy, we also isolated the mutant F1SX39 phage (Fig. S4A), the genome of which contains *gp19* that equally encodes a small tail protein with mutations (Fig. S4B). Subsequently, the effects of four mutant genes on the ability of A1HA147 to infect *mepM-*KO strains were determined. As shown in Fig. 4(C), after inducing *gp25, gp67*, or *gp74* expression, separately, the A1HA147 phage still could not induce the formation of clear plaques on the *mepM-*KO strain-containing plate. On the contrary, upon induction of mutant *gp34* expression, plaque formation could be observed on similar plates. Although the plaques were relatively vague, their presence indicated that mutant *gp34* expression could partially rescue the defective phenotype of the A1HA147 phage in relation to *mepM-*KO strain infection. Similarly, F1SX39 mutant *gp19* expression in the *mepM-*KO strain could also reverse the resistance of the strain to phages (Fig. S4C).

To identify the key amino acid mutation sites in the Gp34 protein of the A1HA147 phage, four residues that underwent evolution were mutated one by one, and the complementary effects of the expression of these individual amino acid residue mutations on phage phenotypic defects in relation to *mepM-*KO strain infection were determined. As shown in Fig. 4(D), after ATc induction, K171L, D445H, and A507V mutant expression did not induce the formation of A1HA147-related plaques on *mepM-*KO strain-containing plates. However, upon expression of the V506E mutation-harboring gene, lighter plaques could be observed on the plates, and this result is in line with the ability of phages to infect MsWT strains expressing the V506E mutation-harboring gene, indicating that A1HA147 Gp34(V506E) can partially restore *mepM-*KO sensitivity to the A1HA147 phage. Similarly, the expression of mutant *gp19* (P379A) of the F1SX39 phage can restore *mepM-*KO sensitivity to the F1SX39 phage (Fig. S4D).

Further, using a homologous recombinant strategy, WT *gp34* was replaced in the phage genome with the mutated gene to obtain the corresponding recombinant A1HA147 phage harboring a single residue mutation (Fig. S4E and G). Strikingly, similar to the A1HA147-Mut phage, the recombinant A1HA147-*gp34* (V506E) phage could induce the formation of plaques and exhibited good lytic activity on the *mepM-*KO strain (Fig. 4(E)). F1SX39-*gp19* (P379A) also induced the formation of clear plaques on *mepM-*KO strain-containing plates (Fig. 4(F) and Fig. S4F). These results indicate that the Gp34 (V506E) mutation in A1HA147 and the Gp19 (P379A) mutation in F1SX39 are responsible for the restoration of the infective ability of the phages towards *mepM-*KO strains.

Therefore, mutations in the phage small tail protein confer infectivity to progeny phages towards the *mepM*-KO strain.

### 2.6 Tail proteins expressed in the *mepM*-KO strain exhibit adsorption defects

**Fig. 5:**
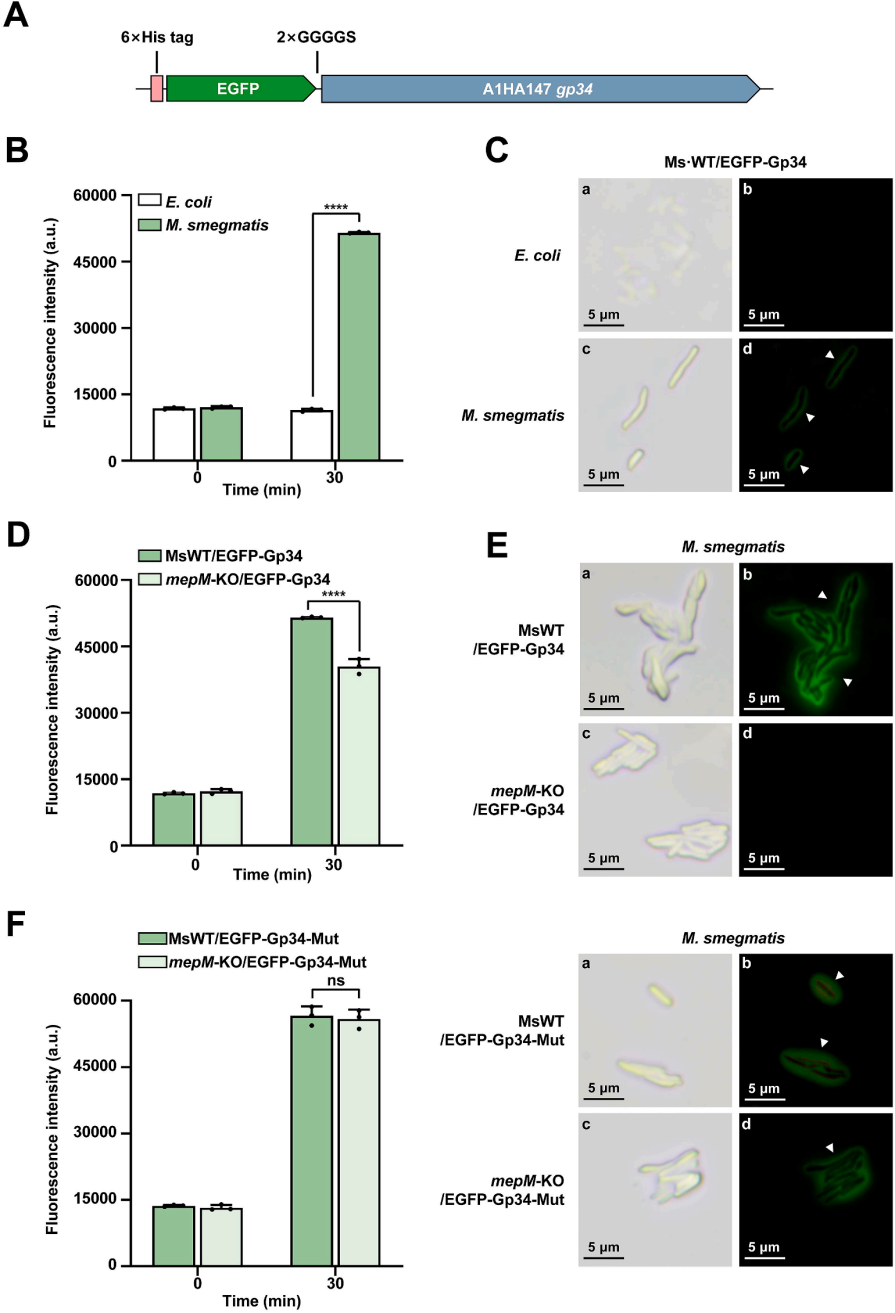
**Tail proteins expressed in the*****mepM*****-KO strain exhibit adsorption defects.** **(A)** Schematic of the ORF structure of the EGFP-fused Gp34 protein. 2 × GGGGS was added as a linker between EGFP and Gp34. **(B)** Quantitative analysis of the association of EGFP-Gp34 with the *E. coli* and *M. smegmatis* cell surface. The EGFP-Gp34 protein was incubated with *E. coli* and *M. smegmatis* for 45 min and subjected to a relative fluorescence intensity assay using an enzyme-linked immunosorbent assay (ELISA) reader. All data in the quantitative analysis are expressed as mean ± standard deviation. Biological replication: *n* = 3. Two-factor analysis of variance was performed using GraphPad Prism for *p*-value calculation. The asterisk indicates a significant difference between two groups (ns indicates no significant difference, ∗∗∗∗ indicates *p* < 0.000 1). **(C)** Fluorescence microscopic assay for EGFP-Gp34 binding to *E. coli* and *M. smegmatis*. Scale bar: 5 μm. **(D)** Quantitative analysis of two distinct EGFP-Gp34 proteins associated with the *M. smegmatis* cell surface. The two fusion proteins, MsWT/EGFP-Gp34 and *mepM*-KO/EGFP-Gp34, expressed in wild-type and *mepM*-knockout *M. smegmatis*, respectively, were incubated with *M. smegmatis* for 45 min and subjected to the relative fluorescence intensity assay as described above. Two-factor analysis of variance was performed using GraphPad Prism for *p*-value calculation. The asterisk indicates a significant difference between the two groups (ns indicates no significant difference, ∗∗∗∗ indicates *p* < 0.000 1). Biological replication: *n* = 3. **(E)** Fluorescence microscopy assays for the binding of MsWT/EGFP-Gp34 and *mepM*-KO/EGFP-Gp34 to *M. smegmatis*. Scale bar: 5 μm. **(F)** Quantitative (left panel) and fluorescence microscopy (right panel) assays for the binding of EGFP-MutGp34 to *M. smegmatis*. Scale bar: 5 μm. (ns indicates no significant difference). Biological replication: *n* = 3.

Small tail proteins typically play a role during the adsorption stage of phage infection. We evaluated Gp34 (small tail protein) expressed by WT and *mepM-*KO strains for differences in their ability to adsorb to the surface of mycobacteria. First, a *gp34* fusion gene with an EGFP-tag at its N-terminus was constructed (Fig. 5(A)), and fused-Gp34 proteins from two different sources were purified. When EGFP-Gp34 from the WT strain (MsWT/EGFP-Gp34) was incubated separately with *E. coli* and *M. smegmatis*, no significant difference in fluorescence intensity was observed between the two species at the starting time. However, after 30 min of incubation, the fluorescence intensity for *M. smegmatis* was significantly higher than that for *E. coli* (Fig. 5(B)). Further fluorescence microscopic observations revealed a clear green fluorescence only on the surfaces of *M. smegmatis* cells, with no surface luminescence observed for *E. coli* (Fig. 5(C)), indicating that the EGFP-Gp34 protein could specifically bind to the surface of *M. smegmatis*. Consistently, similar specific binding phenomena were observed when Gp34 was labeled with fluorescein 5-MF (Fluorescein-5-maleimide) (Fig. S5A and B).

Subsequently, the EGFP-containing fusion protein was isolated from the *mepM-*KO strain (*mepM*-KO/EGFP-Gp34), and MsWT/EGFP-Gp34 and *mepM*-KO/EGFP-Gp34 were separately incubated with *M. smegmatis* for 30 min. The fluorescence intensity for *M. smegmatis* incubated with *mepM*-KO/EGFP-Gp34 was found to be significantly lower than that for *M. smegmatis* incubated with MsWT/EGFP-Gp34 (Fig. 5(D)). Furthermore, microscopic observations revealed a green fluorescence only on the surfaces of *M. smegmatis* cells co-incubated with MsWT/EGFP-Gp34 in the fluorescence field, with only a weak green fluorescence observed for the mycobacterial strain co-incubated with *mepM*-KO/EGFP-Gp34 (Fig. 5(E)). Following labelling with fluorescein 5-MF, the ability of Gp34 purified from the *mepM-*KO strain to adsorb onto the mycobacterial surface was confirmed to be significantly impaired (Fig. S5C and D). Similarly, when the MutGp34 mutant from the evolution-derived A1HA147-Mut phage was labeled with EGFP or fluorescein 5-MF, the mutant isolated from both the *mepM-*KO and WT strains showed clear binding to the surface of *M. smegmatis* (Fig. 5(F) and Fig. S5E).

These results indicate that the phage tail protein, Gp34, can specifically adsorb onto *M. smegmatis*, and that *mepM*-KO strain-derived Gp34 exhibits significant adsorption defects when compared to that derived from MsWT strains.

### 2.7 Tail proteins undergo MepM-dependent methylation-based modification in mycobacteria

**Fig. 6:**
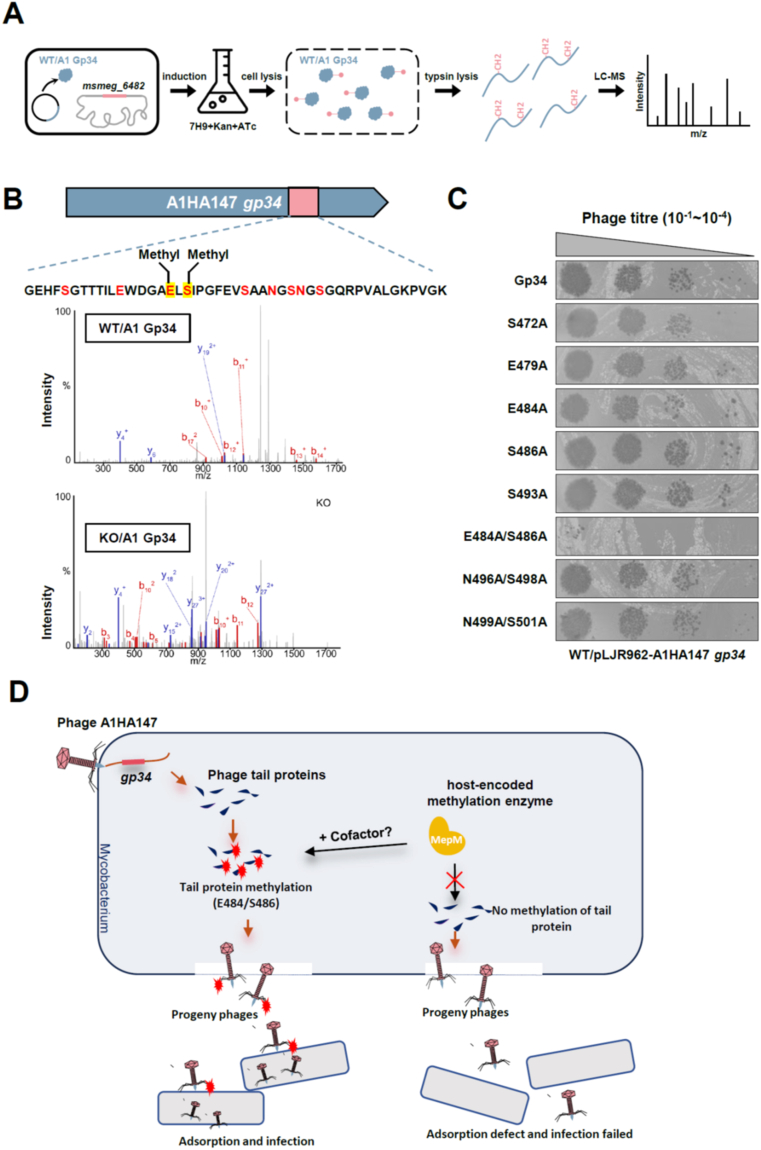
**MepM-dependent methylation-based modification of tail proteins is essential for progeny phage infectivity.** **(A)** Schematic of post-translational modifications (PTMs) for mass spectrometric detection of A1HA147-Gp34 expressed in different bacterial strains. Using trypsin, A1HA147-Gp34 expressed in wild-type and *mepM*-KO strains was digested into peptide segments of different sizes for protein PTM detection through LC-MS. **(B)** Liquid chromatography-mass spectrometry-based analysis of PTMs in the A1HA147-Gp34 protein. Amino acids that may undergo modification are marked in red and are shown in the upper panel, and both E484 and S486 are methylated. The lower panel shows the LC-MS secondary mass spectrum of Gp34 from different bacterial strains. WT/A1-Gp34 represents the secondary mass spectrum of the peptide segment, GEHFSGTTILEWDGAELSIPGFEVSAANGSRGSGQRPVGK, derived from Gp34 expressed in wild-type *M. smegmatis*; KO/A1-Gp34 represents the secondary mass spectrum of the same Gp34 peptide segment expressed in the *mepM*-KO strain. **(C)** Determination of the second-round infection efficiency for A1HA147-Gp34 and its mutants. A1HA147 Δ *gp34* and Gp34 recombinant expression was induced using the MP buffer, and the culture solution was filtered to collect the progeny phages produced during the second round of infection, induced by 200 ng/mL ATc. The infection efficiency of second-round phages was subsequently determined using double-layered plates. **(D)** Infection model of the progeny phages promoted by Gp34 methylation in *M. smegmatis*. Methylation-based modification of the phage tail protein encoded by the *gp34* of A1HA147 is essential for progeny infectivity. The modification is directly or indirectly dependent on the methyltransferase, MepM, in *M. smegmatis*. When the phage infects a *mepM*-deleted strain, the tail protein Gp34 loses specific modifications, which inhibits its binding to the bacterial surface and leads to loss of progeny infectivity.

Gp34 expressed in strains lacking the methyltransferase-encoding gene, *mepM*, exhibits defects in adsorption onto mycobacteria, and this subsequently affects progeny phage infectivity. It is speculated that Gp34 may require methylation under the action of MepM to exhibit normal adsorption activity. To confirm this hypothesis, two distinct Gp34 proteins isolated from MsWT and the *mepM-*KO strain were digested by trypsin and subsequently analyzed through liquid chromatography-mass spectrometry (LC-MS) (Fig. 6(A) and Fig. S6A). In total, 45 peptide segments with different PTMs were identified in Gp34 isolated from the WT strain (WT/A1Gp34) (Fig. S6B), while only 33 peptide segments with different PTMs were identified in that isolated from the *mepM-*KO strain (KO/A1Gp34) (Fig. S6C). Of note, a peptide segment covering the mutated Gp34 V506 amino acid site was identified in both the WT/A1Gp34 and KO/A1Gp34 proteins, i.e., GEHFSGTTTILEWDGAELSIPGFEVSAANGSNGSGQRPVALGKPVGK (Fig. 6(B)). Three methylation-based modifications and one glycosylation-based modification, i.e., 1xHexNAc [N/S/T] and 3xMethyl [D/E/N/Q/R/S/T], were detected in the identified peptide segment in WT/A1Gp34. However, only one methylation-based modification, 1xMethyl [D/E/I/L/S], was detected in the peptide segment identified in KO/A1Gp34, with no glycosylation-based modification detected. Based on peptide mass spectrometric information, certain residues of WT/A1Gp34, including S472, E479, E484, S486, S493, S498, and S501, probably underwent additional methylation-based modifications that lack in KO/A1Gp34. Additionally, the N496 and/or N499 residues may contain glycosylation-based modifications (Fig. 6(B)).

Therefore, our results indicate that MepM-dependent methylation- and glycosylation-based modifications occur in the peptide segments of the phage small tail protein, Gp34.

### 2.8 MepM-dependent methylation modification of tail proteins is essential for the infectivity of progeny phages

To further confirm the correlation between these Gp34 PTMs and progeny phage infectivity, A1HA147 Δ *gp34* lysogenic *M. smegmatis* was produced, and the expression of genes encoding Gp34 associated with a series of PTM-related amino acid point mutations was induced in the lysogenic bacteria. As shown in Fig. S6D, after inducing *gp34* expression in the lysogen, a large number of clear, transparent plaques were observed on the double-layered plate, indicating that WT Gp34 expression through the vector could effectively complement infectivity in mutant phages lacking this gene in their genome. Similar to *gp34*, several mutant genes encoding proteins with the S472A, E479A, S486A, S493A, N496A/S498A, or N499A/S501A mutations could also complement mutant phage infectivity; in addition, the number of plaques on the double-layered plates was not significantly different from that observed with WT *gp34*. These results indicate that these mutations had no significant effect on phage infectivity (Fig. S6D), suggesting that the mutant residues could not undergo MepM-based PTM. However, the expression of Gp34 containing the E484A or Gp34-S486A mutation could not restore the A1HA147 Δ *gp34* phage infectivity defect, with plaques formed on the double-layered plate being significantly smaller and more turbid. Particularly, following the expression of Gp34 containing the E484A/S486A dual mutation, the number of plaques formed on the double-layered plate significantly decreased, with only a few single plaques observed (Fig. S6D). This indicates that the single amino acid mutation, E484A or S486A, in A1HA147 Gp34, could weaken the infectivity of the phages towards *M. smegmatis* to some extent. Strikingly, dual mutations in Gp34 containing E484A/S486A could almost completely abolish phage infectivity towards *M. smegmatis*, suggesting that the PTM of these two amino acid residues is critical for conferring infectivity to the phages.

Further, double-layered plates containing a mixture of the A1HA147 Δ *gp34* lysogenic strain and a WT Gp34 recombinant-expressing strain were prepared, and the potency of progeny phages produced during subsequent rounds of infection was determined. As shown in Fig. 6(C), after diluting the phage filtrate 10^4^-fold, progeny phages generated following the induction of A1HA147 Gp34 expression, as well as that of its mutants containing the S472A, E479A, S486A, S493A, N496A/S498A, or N499A/S501A mutations, produced plaques on double-layered plates, with the number of plaques not being significantly different from that observed in the control group. By contrast, progeny phages generated in the presence of the Gp34-E484A/S486A double mutant produced plaques only after a 10^2-fold dilution of the phage lysate, and the plaque number was markedly reduced compared with that of the WT control and the other mutant groups (Fig. 6(C)). These results indicate that dual E484A/S486A mutations in Gp34 markedly impairs progeny phage multiplication in subsequent rounds of infection, resulting in an approximately 10^3-fold reduction in infectivity.

Next, we determined the extent to which these potential PTM site mutations affected infection efficiency in subsequent rounds of infection. As shown in Fig. S6E, when the expression of WT Gp34 and Gp34 mutants containing the S472A, E479A, S486A, S493A, N496A/S498A, or N499A/S501A mutations was induced in lysogenic bacteria, approximately 7 × 10^7^ plaques were produced by progeny phages following subsequent rounds of infection, with no significant difference observed between these plates and control plates. However, upon induction of Gp34-E484A/S486A expression, progeny phages produced only approximately 1.6 × 10^4^ plaques (Fig. S6E). These results indicate that mutations at two PTM sites (E484A/S486A) in A1HA147 phage Gp34 led to a significant decrease in the number of plaques produced by progeny phages during subsequent rounds of infection.

Collectively, MepM-dependent methylation-based modification of the two Gp34 residues, E484 and S486, in mycobacteria is necessary for progeny phage infectivity.

## 3 Discussion

Although PTMs are important mechanisms for viral survival within host cells, it is unclear whether phage protein methylation occurs; in addition, the potential effects of PTMs on phage infection are unclear. In this study, we found that methylation-based modification of the phage tail protein is essential for progeny virus infectivity and that this type of PTM in *M. smegmatis* is directly or indirectly dependent on the widely conserved methyltransferase, MepM. When the A1HA147 phage infects a *mepM*-deficient mycobacterial strain, the ELS amino acid motif of its tail protein, Gp34, loses specific methylation modifications, which inhibits its binding to the surface of *M. smegmatis* and leads to loss of infectivity in progeny phages (Fig. 6(D)). Our findings provide original experimental evidence of the previously undefined mechanism by which bacteriophages utilize host bacteria-encoded enzymes to methylate their proteins for infectivity maintenance.

The most important finding of this study is that a bacterium-derived methyltransferase is essential for the phage infectivity through directly or indirectly affecting PTM of phage tail proteins. During infection, viruses often exploit host machinery for PTM of their proteins. For instance, the Vif protein of HIV-1 hijacks the Cullin5 E3 ubiquitin ligase complex of the host to degrade the antiviral factor, APOBEC3G (Yu et al., 2003). During infection of host mycobacteria, the TM4 mycobacteriophage utilizes the host serine/threonine kinase, StpK7, to phosphorylate and suppress the expression of genes in the BREX-like system, thereby promoting phospholipid synthesis and enhancing phage adsorption (Li et al., 2023). *E. coli* T5 and T6 phages can modify their tail-fiber and receptor-binding proteins via host tRNA-dependent aminoacylation to alter adsorption specificity or resist host antiphage factors (Al-Shayeb et al., 2020). However, there have been no reports on phage protein modification by host methyltransferases. To date, only sporadic studies have documented the role of protein methylation in the interaction between eukaryotic viruses and their hosts. Our findings, combined with reports on eukaryotic viruses, suggest that methylation-based PTMs may represent a conserved mechanism for the regulation of both prokaryotic and eukaryotic viral infections. Nonetheless, an intriguing phenomenon is that we cannot clearly detect the methylation activity of MepM using purified proteins *in vitro*, suggesting that within mycobacterial cells, the MepM protein may collaborate with other unidentified cofactors to accomplish this post-translational modification. Given that there are multiple methylation-related genes near the *mepM* gene in the genome of *M. smegmatis*, it is most likely that MepM forms a functional complex with one or several proteins encoded by these methylation-related genes to jointly accomplish the methylation modification, which requires further investigation.

During phage infection, methyltransferases catalyze the methylation reactions commonly involved in the epigenetic modification of nucleic acids. These reactions can contribute both to promoting bacterial host defenses against viruses and aiding phage evasion of host immunity (Gong et al., 2024; Martinez-Cazorla et al., 2025; Takahashi et al., 2025). Interestingly, in this study, we found that mycobacteriophages require the host-encoded methyltransferase, MepM, for PTM of their own proteins during infection, through which they maintain infectivity. Of note, our evolutionary conservation analyses revealed that the MepM domain is widely distributed in the genomes of archaea, bacteria, and eukaryotes, and highly conserved in methytransferase encoded by different viruses in both prokaryotes and eukaryotes, suggesting that the protein may originate from bacteriophages or viruses. During the evolutionary process of viruses, some of their genes may have been integrated into the host genome. Among these are endogenous viral elements (EVEs), which are genetic components formed by the complete or partial integration of ancient viral genomes into the genome of another organism; EVEs are widely distributed in plant and animal genomes (Aiewsakun & Katzourakis, 2015). The presence of EVEs, such as the Bornavirus-like element (EBLL) (Horie et al., 2016), which is retained in bat genomes, and Bornavirus-like nucleoprotein elements (Fujino et al., 2014), retained in mammalian genomes, often confers resistance to exogenous viral infections. During infection, bacteriophages also integrate their genes into the host genome, and this system is referred to as the phage-encoded ESX secretion toxin system (PEST); the integrated genes are referred to as phage-induced chromosomal islands (PICIs) (Alqurainy et al., 2023; Fillol-Salom et al., 2022), and their expression induces resistance to exogenous phage infections. However, there are no reports on the functioning of EVEs or PICIs as host factors for phage infection. In this study, we identified the widely conserved MepM-encoding gene as a host factor, with phage infection critically relying on its function. We speculate that mycobacterial *MepM* is a residual DNA fragment from ancient phages that acts as a key host factor for facilitating phage invasion.

Phage tail proteins play a crucial role in the infection process by participating in host receptor recognition or facilitating binding (Hu et al., 2020; Leprince & Mahillon, 2023; Nobrega et al., 2018), facilitating host cell wall or cell membrane penetration, and promoting DNA injection (Xu et al., 2016). Gp34, encoded by the phage identified in this study, is a tail protein that can specifically bind to the surface of host mycobacteria. Through evolutionary screening, we identified residue V506 in Gp34 as a potential key target for attack by defense proteins in methylation-deficient bacterial strains, suggesting that the V506E mutation may help the phage evade the defense system of knockout strains, as well as attacks by the host. Additionally, our mass spectrometric analysis and site-directed mutagenesis experiments demonstrated that amino acid residues E484 and S486 in Gp34 are critical sites for methylation-based modification by mycobacterial methyltransferases. Methylation-based modifications at these two sites can confer on the phage the ability to adsorb to the surface of host bacteria for successful infection. Although this mutation-driven viral escape mechanism is similar to that previously reported for other phages (Li et al., 2025), the specific mechanism by which methylation-based modification of Gp34 alters its interaction with host bacterial surface recognition receptors to evade host defenses is yet to be elucidated.

In summary, this study showed that methylation-based modification of phage tail proteins depending on a conserved host protein (MepM) confers the infectivity of progeny viruses. These findings reveal a novel, hitherto undescribed mechanism for viral infection, enriching our understanding of the complexities of host-phage interactions.

## 4 Materials and methods

### 4.1 Bacterial strains, phages, and cultivation conditions

The bacterial strains and bacteriophages used in this study are listed in Table S1. *M. smegmatis* mc^2^155 strains were cultured on Middlebrook 7H10 agar (BD Difco, USA) supplemented with 0.5% glycerol and 10% OADC or in 7H9 medium (BD Difco, USA) supplemented with 0.2% glycerol and 0.05% Tween 80 (Sigma-Aldrich, Germany). *E. coli* strains were cultivated in Luria-Bertani (LB) medium. All strains were incubated at 37 °C, and liquid cultures were shaken at 160 rpm. The mycobacterial phages used in this study were isolated from geographically diverse environmental samples in China and were propagated using WT *M. smegmatis* mc^2^155 strains.

### 4.2 Plaque assay for phage infectivity

Phage infectivity was evaluated via a double-agar overlay plaque assay following previously described protocols. Briefly, the phage solution was serially diluted tenfold, and 2 μL of the diluted solution was spotted onto soft agar overlays containing mid-log-phase *M. smegmatis* mc^2^155 (OD_600_ = 1.0). After incubation overnight at 37 °C, plaques were counted for the calculation of infectivity efficiency, expressed as plaque-forming units per milliliter (PFU/mL).

### 4.3 Scarless gene knockout in *M. smegmatis*

Gene knockout was performed following previously described procedures with significant modifications (Liu et al., 2014). A 1 000 bp upstream and downstream region of the gene to be knocked out was inserted into the pMind-*lacZ-sacB* vector (Table S2 and S3) to obtain a scarless knockout plasmid. Then, the recombinant plasmid was electroporated into *M. smegmatis* mc^2^155 (MsWT) and cultured in a medium containing antibiotics and X-gal for 3∼4 days. After plating, blue single colonies were selected and cultured in liquid medium. Under sucrose counter-selection pressure, the strain underwent double crossover. White single colonies obtained following streaking were cultured and sequenced in antibiotic-free 7H9 medium.

### 4.4 Construction of recombinant mycobacterial strains

Recombinant strains were constructed following previously described procedures with significant modifications (Wetzel et al., 2021). Briefly, the full-length gene, *MSMEG_6482*, or other gene fragments were cloned into a site between the *Cla*I and *Not*I restriction enzyme sites of the pLJR962 vector (Addgene, USA). The recombinant plasmids were transformed into the *M. smegmatis* mc^2^155 strain and cultured for 3 days on 7H10 medium containing 25 μg/mL kanamycin. For the induction of gene expression in lysogenic bacteria, the *gp34* point mutants of the A1HA147 phage were cloned into pLJR962 and transformed into the *M. smegmatis* mc^2^155 lysogenic strain derived from the A1HA147 Δ*gp34* phage. After culturing for 3 days on 7H10 medium containing 25 μg/mL kanamycin, single colonies were selected for further induction and expression.

### 4.5 Structural homology search and Last Common Ancestor (LCA) analysis

Structural homology searches were performed using Foldseek (Kempen et al., 2024). The afdb and bfvd databases were downloaded from “https://foldseek.steineggerlab.workers.dev.” The alignment type was set to TMalign (--alignment-type 1), alignment coverage to bidirectional (--cover-mode 0), target coverage to 0.8 (-c 0.8), and the TM-score threshold to 0.5 (--tmscore-threshold 0.5). Prefiltration was not implemented, and an all-vs-all alignment was conducted (--exhaustive-search). An LCA (Last Common Ancestor) analysis was conducted on the best-matching proteins using the MMseqs2 LCA module (Steinegger & Söding, 2017). The resulting LCA data were visualized using Pavian (Breitwieser & Salzberg, 2020) for the generation of Sankey plots.

### 4.6 Phage adsorption assays

Assays were performed following previously described procedures with modifications (Barsom & Hatfull, 1996). Briefly, mid-log-phase *M. smegmatis* mc^2^155 (OD_600_ ≈ 1.5) was harvested, washed, and resuspended in Tween 80-free 7H9 medium to an OD_600_ of 1.0. Triplicate bacterial suspensions (10 mL) were infected with the phage solution at an MOI of 0.1, with a phage-only control included. After static incubation at 37 °C, samples were collected at specified intervals and filtered immediately (0.22 μm); then, the filtrates were titered using a plaque assay to determine the concentration (PFU/mL) of phages in the samples not absorbed by *M. smegmatis*.

### 4.7 Phage genome injection analysis

The genome injection efficiency of the A1HA147 or F1SX39 phage was evaluated through a transfection-based infectivity assay (Li & He, 2026; Mohammed et al., 2023). Purified phage genomic DNA was electroporated into *M. smegmatis* mc^2^155. After a 1 h resuscitation period in 7H9 broth at 37 °C with shaking, the cells were mixed with 1 mL of a mid-log-phase culture (OD_600_ = 1.0) and embedded in soft agar overlays on 7H10 plates. Plaques were counted after 48 h of incubation at 37 °C, and the transfection titer (PFU/mL) was calculated to determine the efficiency of phage genome injection and subsequent replication.

### 4.8 Phage genome replication assay

Phage genome replication was assessed as previously described (Tal et al., 2022). Briefly, mid-log-phase *M. smegmatis* mc^2^155 was harvested and resuspended in triplicate in 10-mL Tween 80-free 7H9 medium aliquots. The suspensions were inoculated with A1HA147 or F1SX39 phages at an MOI of 0.1 and incubated statically at 37 °C for 30 min to allow for adsorption. Thereafter, the cells were collected and resuspended in 100 mL of fresh Tween 80-free medium. At specified time points post-inoculation, total genomic DNA was isolated using a commercial kit (Aidlab, China). Phage DNA copy number was determined through qPCR and normalized to the host 16S rRNA gene.

### 4.9 Quantitative Reverse Transcription PCR (RT-qPCR)

Quantitative Reverse Transcription PCR-based gene expression assays were performed as previously described (Barman et al., 2022). Briefly, *M. smegmatis* was cultured in 7H9 medium to an OD_600_ of 1.0. Then, the bacterial cells were collected through centrifugation and resuspended in 7H9 medium without Tween 80. Next, bacteriophages were added to the cultures at an MOI of 0.1, and the cultures were incubated at 37 °C on a shaker. Samples were collected at 0, 30, 90, and 150 min post-adsorption for both the WT and *mepM*-KO strains. mRNA was extracted using an RNA extraction kit (Aidlab, RN43), and reverse-transcribed into cDNA using a reverse transcription kit (Aidlab, PC1802). The expression levels of phage genes in the bacterial samples were determined at different time points using a real-time fluorescence quantitative PCR detection kit (Aidlab, PC3301). All gene expression levels were normalized using *M. smegmatis sigA* as an internal reference. All data are presented as mean ± standard deviation (*n* = 3, biological replicates).

### 4.10 Growth curve evaluation of mycobacteria under phage infection

The growth curve for *M. smegmatis* was generated following a previously described method with slight modifications (Ofir et al., 2021). Bacterial cells (OD_600_ = 1.0) were centrifuged and collected. The initial OD_600_ for WT or *mepM*-KO strain bacterial cultures was set at 0.2, with MOIs of 5 or 0.05 for phage infection. Then, the cultures were shaken at 37 °C in Tween-free 7H9 medium. Samples were collected at 1 h intervals from 0 to 8 h post-infection and at 4 h intervals from 8 to 28 h post-infection, and compared with uninfected strain samples. All data are presented as mean ± standard deviation (*n* = 3, biological replicates).

### 4.11 Mycobacteriophage evolution assays

Mycobacteriophage evolution experiments were performed following previously described procedures with modifications (Li et al., 2024). A phage enrichment solution at a titer of approximately 10^6^ was diluted and spotted on a double-layered medium containing *mepM*-KO, and this resulted in the formation of faint, poorly defined plaques caused by infection with A1HA147 or F1SX39 at a high titer. These plaques were picked and resuspended in MP buffer. After lysis and filtration, the solution was mixed with the *mepM*-KO bacterial culture and co-incubated at 37 °C for 48 h. Then, the filtrate was poured onto a double-layered plate containing *mepM*-KO and incubated at 37 °C for 24 h; thereafter, the MP buffer was added to induce a round of phage evolution. This process was repeated through multiple rounds of evolution, ultimately yielding the mutant phage, which could form clear plaques on the double-layered plate containing the *mepM*-KO strain. Finally, single plaques were selected for enrichment culture and then genomic sequencing.

### 4.12 Construction of recombinant mycobacteriophages

Recombinant mycobacteriophages were constructed following a previously described procedure with modifications (Liu et al., 2014). The *M. smegmatis* strain was cultured in 7H9 medium to an OD_600_ of 1.0, and bacteriophages were added to induce lysogeny. Subsequently, the bacteria were streaked on 7H10 medium for isolation, and single colonies were picked and verified via PCR. For recombinant phage construction, A1HA147 *gp34* was first deleted using seamless knockout technology to generate the A1HA147 Δ*gp34* phage, which was subsequently used to infect the *M. smegmatis* strain to obtain its lysogenic Msm strain (A1HA147 Δ*gp34*). Next, the point-mutated gene fragment, A1HA147 *gp34* (V506E), was constructed and inserted into the *gp34*-deleted site of the lysogenic Msm strain (A1HA147 Δ*gp34*), resulting in the obtention of the lysogenic phage strain, (A1HA147 *gp34* (V506E). Finally, a double-layered plate was prepared using the lysogenic strain, and after 24 h of incubation, 5 mL of MP Buffer (50 mM Tris, 150 mM NaCl, 10 mM MgSO_4_, 2 mM CaCl_2_) was added to it for soaking. Then, the mixture was slowly shaken at 100 rpm for 24 h at 16 °C. The supernatant containing the A1HA147 *gp34* (V506E) phage was filtered through a 0.22 μm membrane, diluted to an appropriate concentration, and spread on a double-layered plate to isolate individual plaques. PCR and sequencing were performed for verification. Similar methods were used to construct the recombinant F1SX39-*gp19* phage and its point-mutated gene variants.

### 4.13 Fluorescence intensity assays for recombinant phage proteins

Fluorescence intensity was determined following previously described procedures with modifications (Beckham et al., 2020). A1HA147 phage *gp34* or its N-terminal fused with an EGFP tag, as well as the gene containing three cysteine residues at its N-terminus, were separately cloned between the *Bam*HI and *Cla*I restriction sites of the pMyNT vector (Addgene, USA). A 2×GGGGS linker was inserted between EGFP and *gp34*. The recombinant plasmids, pMyNT-Gp34, pMyNT-EGFP-Gp34, pMyNT-Cys_3_-Gp34, pMyNT-EGFP-Mut-Gp34, and pMyNT-Cys_3_-Mut-Gp34, were consequently constructed; these plasmids were then transformed into WT or *mepM*-KO strains for expression. Next, the cultures were shaken at 37 °C until an OD_600_ of 1.0 was attained; this was followed by induction with 0.2% acetylamine for 24∼36 h. Thereafter, the cells were collected and subjected to pressure disruption, and their proteins were purified using nickel affinity chromatography. The eluted proteins were dialyzed in a low-salt buffer (20 mM Tris-HCl, 100 mM NaCl, 1 mM DL-dithiothreitol, 10% glycerol) for 1 h and stored at −80 °C. Protein concentration was determined using the Coomassie brilliant blue method. EGFP-Gp34 purified from the WT strain was incubated with *E. coli* and *M. smegmatis* for 45 min. The fluorescence intensity of bacterial strain-bound EGFP-Gp34 was quantified using a microplate reader at excitation and emission wavelengths of 488 and 507 nm, respectively. The Cys_3_-Gp34 protein purified from the WT strain was labeled with fluorescein 5-MF *in vitro*, enabling detection at Ex/Em = 494/518 nm. 5-MF-labeled Cys_3_-Gp34 was incubated with *E. coli* and *M. smegmatis* for 45 min, and its fluorescence intensity upon binding with bacterial strains was quantified using a microplate reader. Similar procedures were used to determine the fluorescence intensities of recombinant F1SX39-Gp19 proteins.

### 4.14 Fluorescence microscopic evaluation

The fluorescent protein, EGFP-Gp34 or 5-MF-labeled Cys_3_-Gp34, was co-incubated with *E. coli* and *M. smegmatis* suspensions at 4 °C for 4 h. After washing the cells thrice with PBS, unbound proteins were removed to reduce background fluorescence. Then, 10 μL of the protein-bacteria solution was mixed with 1∼2 drops of a quenching inhibitor for microscopic observation.

### 4.15 LC-MS/MS analysis of protein modification

Post-translational modification of the phage protein, Gp34, was evaluated using a previously established liquid chromatography-mass spectrometry-based method (Yan et al., 2024) with modifications. The A1HA147 phage *gp34* fragment was cloned into the pMyNT vector and transformed into both wild-type *M. smegmatis* and a *mepM*-knockout strain. The expressed His-tagged Gp34 fusion protein was subsequently purified using a Ni-NTA affinity column. The purified protein was separated by SDS-PAGE. The corresponding gel bands were sliced and reduced with 10 mM dithiothreitol (DTT) at 90 °C for 5 min, alkylated with 20 mM iodoacetamide (IAA) at room temperature for 15 min in the dark, and incubated with trypsin at 37 °C overnight. The digested peptides were desalted with C18 Stages Tips, dried and reconstituted with 1% formic acid for LC-MS/MS analysis. The digested tryptic peptides were analyzed using a Vanquish Neo UHPLC coupled to an Orbitrap Explores 480 mass spectrometer (Thermo Fisher Scientific). The peptides were separated with a binary buffer system on an in-house prepared capillary column (75 μm × 150 mm) packed with 1.9-μm, 120 Å ReproSil-Pur C18-AQ silica particles. Buffer A was 0.1% formic acid in 100% H_2_O and Buffer B was 0.1% formic acid in 80% acetonitrile with 20% H_2_O. Data were acquired in a data-dependent acquisition mode. The peptide identification was performed using Protein Discoverer (Thermo Fisher Scientific) with a mass tolerance of 10 ppm for precursor ions and 0.02 Da for MS/MS fragment ions, allowing for a maximum of two missed cleavages. Identified peptides were filtered to achieve a false discovery rate of less than 1%. Corresponding peptide fragment spectra were manually checked using Thermo Xcalibur 4.1 software. Peptide sequences and modification sites were determined by comparing the acquired secondary mass spectra with theoretical spectra in the database, applying a confidence threshold of >95%. This analysis enabled the identification of various post-translational modification sites on Gp34, including phosphorylation, acetylation, methylation, and glycosylation.

### 4.16 Quantitative and statistical analyses

GraphPad Prism 8 was used for statistical analysis, with data presented as mean ± standard deviation (*n* = 3). The statistical testing methods and significance criteria used are indicated in each legend.

## CRediT authorship contribution statement

**Sijia Fan:** Conceptualization, Data curation, Formal analysis, Investigation, Methodology, Supervision, Validation, Writing – original draft, Writing – review & editing. **Junlong Li:** Conceptualization, Data curation, Formal analysis, Investigation, Methodology, Supervision, Validation, Writing – original draft, Writing – review & editing. **Yuyun Wei:** Data curation, Investigation, Methodology. **Yuxuan Zhang:** Investigation, Methodology. **Xiao Guo:** Data curation, Formal analysis, Software. **Di Wu:** Data curation, Investigation, Methodology, Writing – review & editing. **Zheng-Guo He:** Conceptualization, Data curation, Formal analysis, Funding acquisition, Methodology, Project administration, Resources, Supervision, Validation, Writing – original draft, Writing – review & editing.

## Declaration of competing interest

The authors declare that they have no known competing financial interests or personal relationships that could have appeared to influence the work reported in this paper.
