# Supplementary material for: Phage tail protein methylation enabling the virus to bind to the surface of bacterial cells is vital for progeny infectivity
Source: Cell Insight. 2026 Jul 1;5(5):100345. doi: 10.1016/j.cellin.2026.100345 (PMC13445384; doi:10.1016/j.cellin.2026.100345)
Supplement: Supplementary file 1 [file mmc1.pdf]

Supplemental files

Phage tail protein methylation enabling the virus to bind to the surface of bacterial cells is vital for progeny infectivity

Sijia Fan[1,2], Junlong Li[1,2], Yuyun Wei[1,2], Yuxuan Zhang[3], Xiao Guo[1,2], Di Wu*[,3], Zheng-Guo He*[,1,2]

[1]College of Life Science and Technology, Guangxi University, Nanning 530004, China.
[2]State Key Laboratory of Virology and BioSafety, Taikang Center for Life and Medical Sciences, TaiKang Medical School (School of Basic Medical Sciences), Wuhan University, Wuhan, 430071, China
[3]State Key Laboratory of Metabolism and Regulation in Complex Organisms, TaiKang Center for Life and Medical Sciences, School of Basic Medical Sciences, Wuhan University, Wuhan, 430071, China

*To whom correspondence should be addressed: School of Basic Medical Sciences, Wuhan University, Wuhan, 430071, China.
Email: hezhengguo2024@whu.edu.cn or dwu@whu.edu.cn
Tel: +86-27-68759222, Fax: +86-27-68759222

List of supplemental files

Figure S1 Characteristics of the *mepM*-KO strain and its antiphage spectrum.
Figure S2 Conserved methyltransferase residues of MepM are essential for F1SX39 phage infection.
Figure S3 *mepM* deletion does not affect the primary infection of F1SX39 but causes infection defects in its progeny phages.
Figure S4 Tail protein mutation restores phage infectivity of F1SX39 toward the *mepM*-KO strain.
Figure S5 Fluorescein-labelled tail proteins expressed in the *mepM*-KO strain exhibit adsorption defects.
Figure S6 Methylation-based modification of Gp34 is essential for progeny phage infectivity.
Table S1 Bacterial strains used in this study.
Table S2 Primers used in this study.
Table S3 Plasmids used in this study.

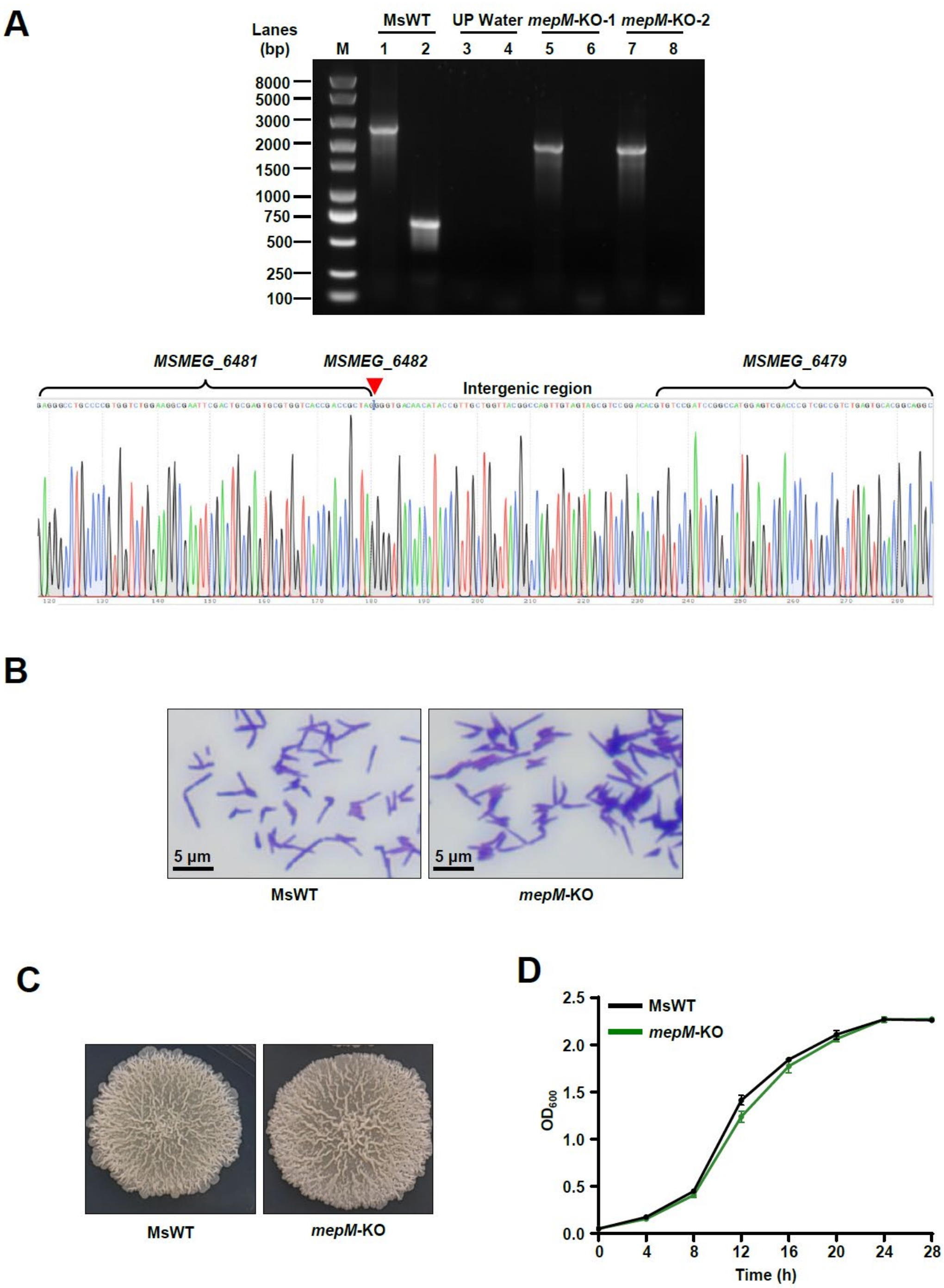
A
Lanes
(bp)
M
MsWT
1
2
UP Water
3
4
mepM-KO-1
5
6
mepM-KO-2
7
8
8000
5000
3000
2000
1500
1000
750
500
250
100
MSMEG_6481
MSMEG_6482
Intergenic region
MSMEG_6479
B
5 μm
5 μm
MsWT
mepM-KO
C
MsWT
mepM-KO
D
MsWT
mepM-KO
2.5
2.0
1.5
1.0
0.5
0.0
OD600
0
4
8
12
16
20
24
28
Time (h)

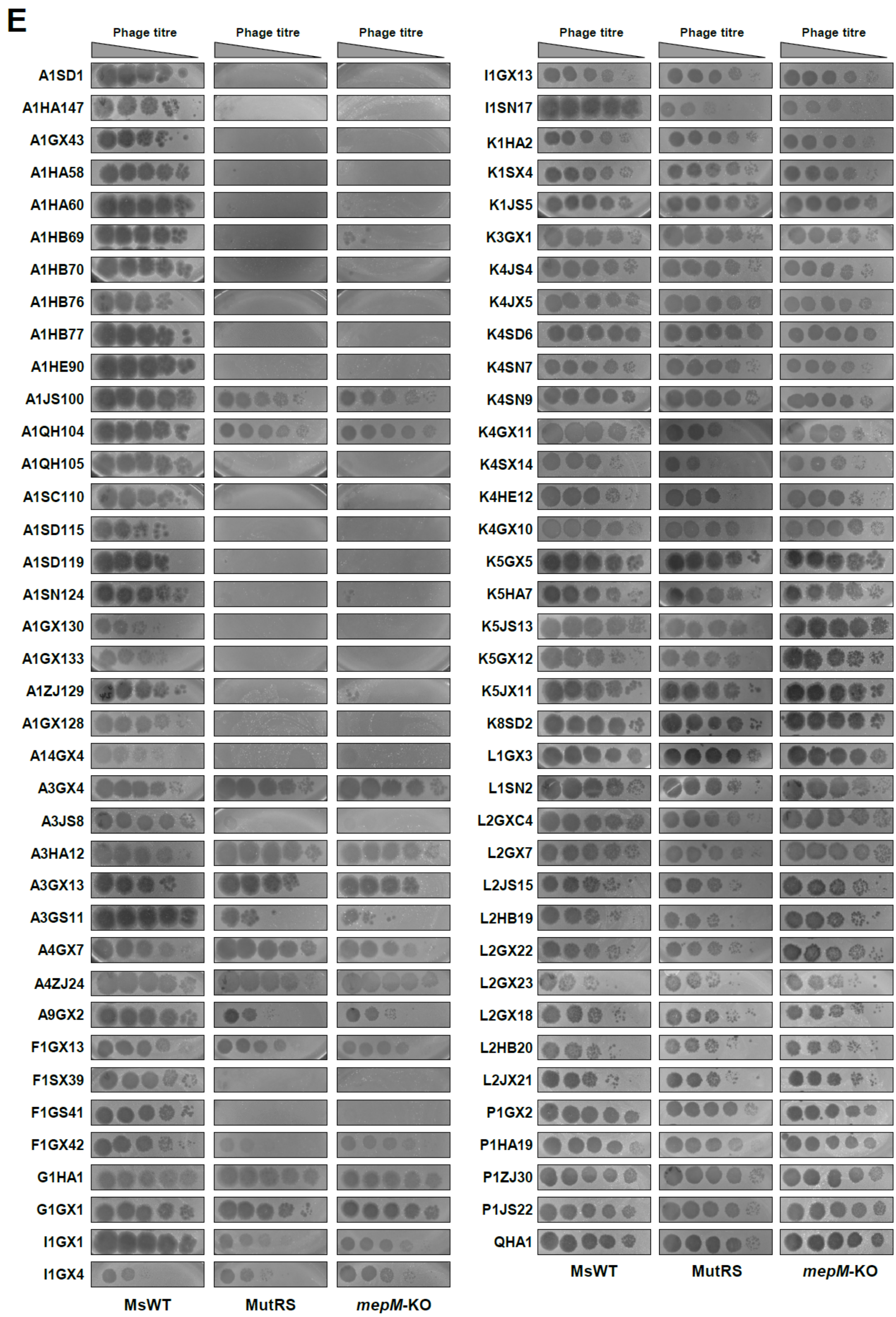

Fig. S1. Characteristics of the *mepM*-KO strain and its antiphage spectrum.
(A) PCR and sequencing validation for *MSMEG_6482* knockout. Lanes 1 and 2 represent DNA bands produced by the wild-type strain, which served as positive controls. Lanes 3 and 4 represent the negative controls. Lanes 5~8 represent the DNA

bands produced by the two correct *mepM*-KO strains amplified using two specific primer pairs.

(B) Morphology of the *mepM*-KO strain. Following staining with 2% crystal violet, bacterial morphology was observed using an optical microscope. Scale bar: 10 μm.

(C) Morphology of the *mepM*-KO strain bacterial colony surface. Wild-type and the *mepM*-KO strains were cultured on 7H10 medium for 3~4 days and used for morphological observation of colonies.

(D) Growth curve of the *mepM*-KO strain. Wild-type and *mepM*-KO strains were inoculated into 7H9 medium and incubated at 37℃ to establish their growth curves. All data are presented as mean ± standard deviation (n = 3, biological replicates).

(E) Determination of the antiphage spectra of the *mepM*-KO and mutant MutRS strains. Different mycobacterial phage subclusters were diluted in a 10-fold gradient to obtain a range of 10^{-1} to 10^{-5}, and each dilution was spotted on double-layered plates containing the wild-type, mutant MutRS, and *mepM*-KO strains for the assessment of plaque formation.

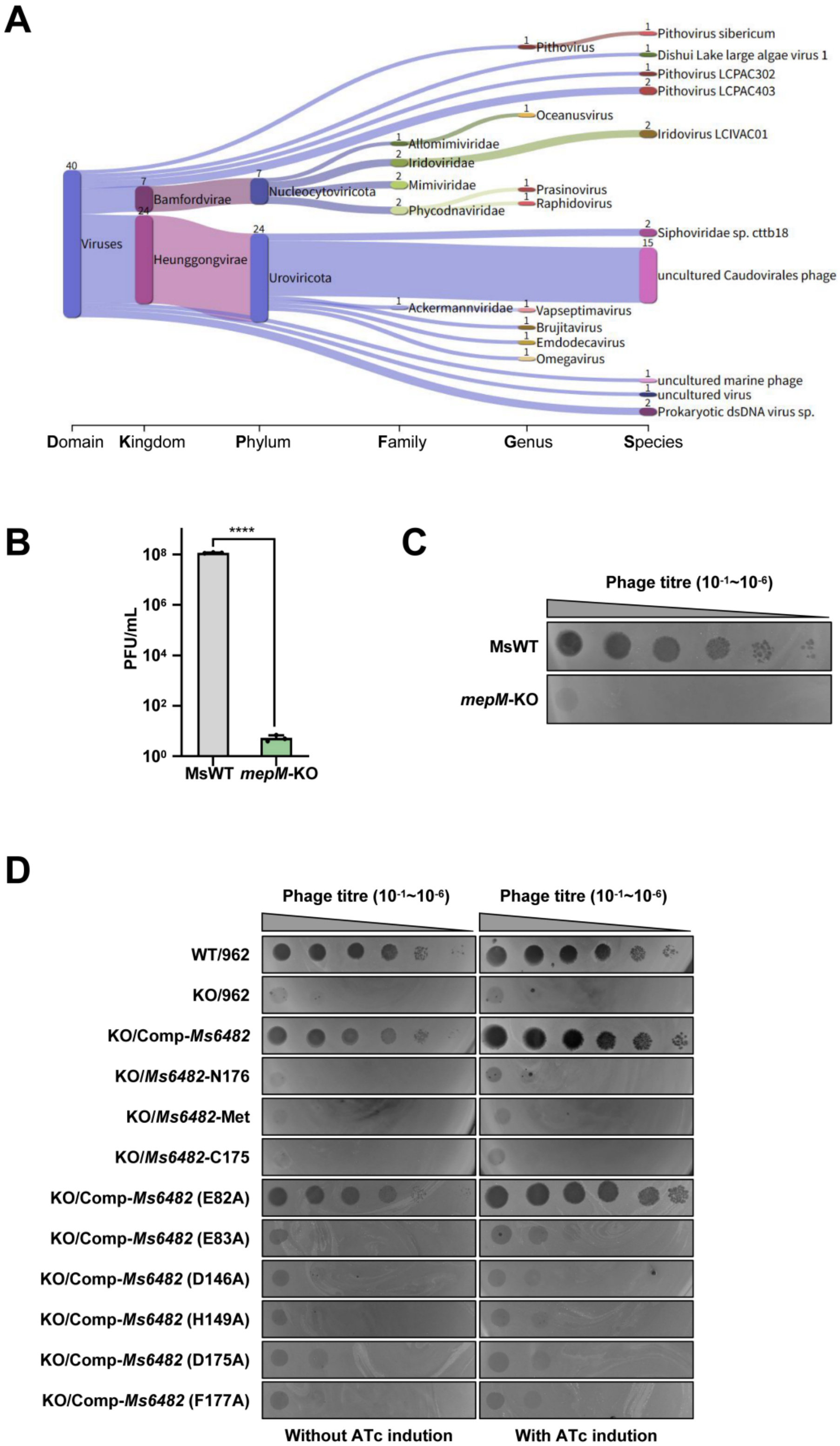
A
40
Viruses
7
Bamfordvirae
24
Heunggongvirae
7
Nucleocytoviricota
24
Uroviricota
1 Allomimiviridae
2 Iridoviridae
2 Mimiviridae
2 Phycodnaviridae
1 Ackermannviridae
1 Pithovirus
1 Oceanusvirus
1 Prasinovirus
1 Raphidovirus
1 Vapseptimavirus
1 Brujitavirus
1 Emdodecavirus
1 Omegavirus
1 Pithovirus sibericum
1 Dishui Lake large algae virus 1
1 Pithovirus LCPAC302
2 Pithovirus LCPAC403
2 Iridovirus LCIVAC01
2 Siphoviridae sp. cttb18
15 uncultured Caudovirales phage
1 uncultured marine phage
1 uncultured virus
2 Prokaryotic dsDNA virus sp.
Domain
Kingdom
Phylum
Family
Genus
Species
B
****
PFU/mL
10^8
10^6
10^4
10^2
10^0
MsWT
mepM-KO
C
Phage titre (10^-1~10^-6)
MsWT
mepM-KO
D
Phage titre (10^-1~10^-6)
Phage titre (10^-1~10^-6)
WT/962
KO/962
KO/Comp-Ms6482
KO/Ms6482-N176
KO/Ms6482-Met
KO/Ms6482-C175
KO/Comp-Ms6482 (E82A)
KO/Comp-Ms6482 (E83A)
KO/Comp-Ms6482 (D146A)
KO/Comp-Ms6482 (H149A)
KO/Comp-Ms6482 (D175A)
KO/Comp-Ms6482 (F177A)
Without ATc indution
With ATc indution

E

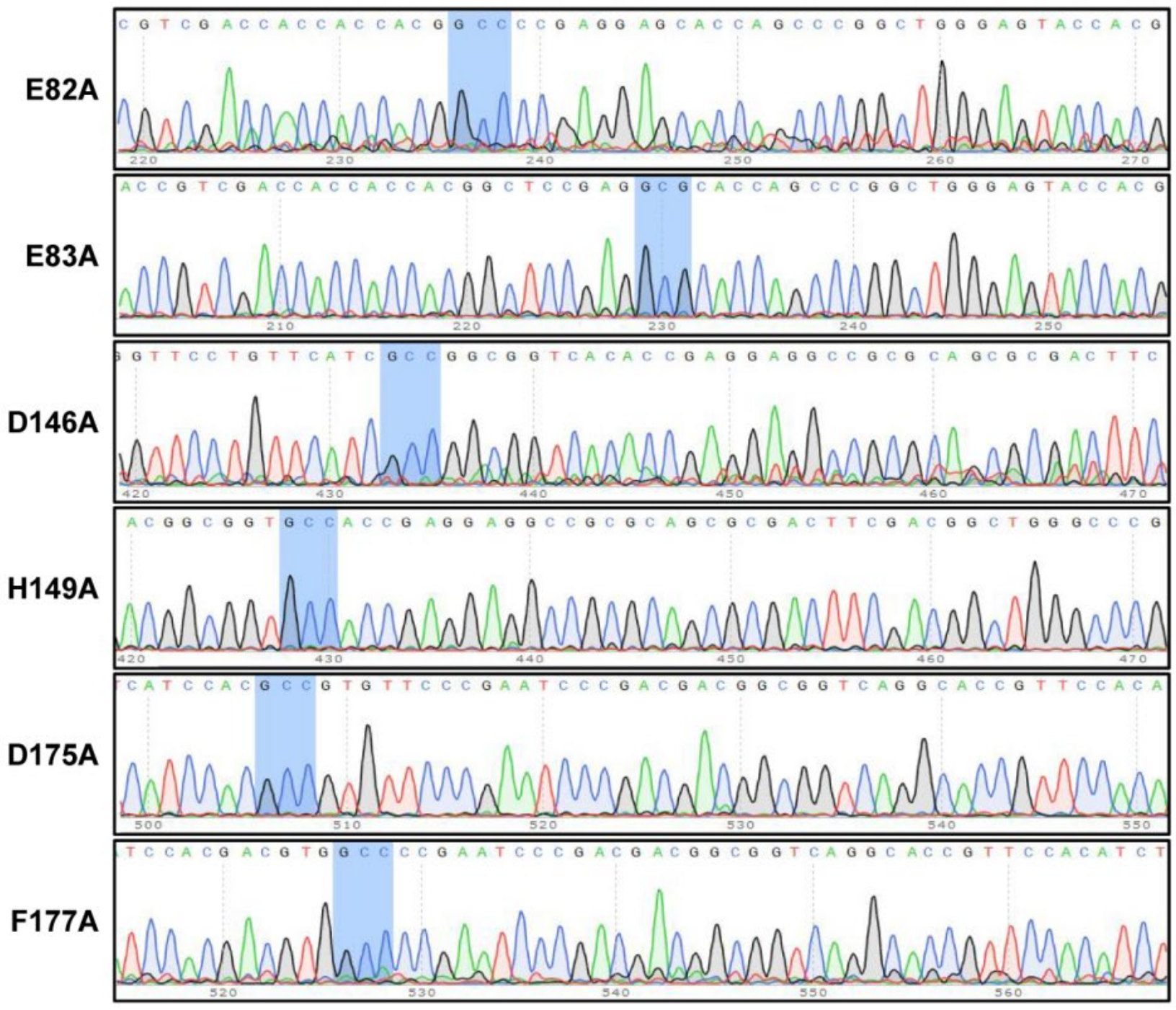

Fig. S2. Conserved methyltransferase residues of MepM are essential for F1SX39 phage infection.

(A) Visualization of the Last Common Ancestors (LCAs) of all BFVD structural homologies of MepM presented as a Sankey plot generated using Pavian. Only the 10 largest taxonomical nodes per rank are displayed.

(B) Effect of *mepM* deletion on the plaque formation efficiency of the F1SX39 phage. F1SX39 was mixed with the *mepM*-KO strain to prepare double-layered plates, cultured for 24 h, and the number of plaques was counted. The error line represents the standard deviation of three repeated experiments. Statistical analysis was conducted using the *t*-test (**** $p < 0.0001$). Biological replication: n = 3.

(C) Comparative assays of the plaque formation ability of the F1SX39 phage in the wild-type and *mepM*-KO strains.

(D) F1SX39 phage infectivity is a function of the methyltransferase activity of MepM. Several truncated genes, such as *Ms6482-N176*, *Ms6482-Met*, and *Ms6482-C175*, as well as point mutant genes containing the E82A, E83A, D146A, H149A, D175A, or F177A mutations, were complemented and expressed in the WT and *mepM*-KO strains. Their effects on F1SX39 phage infectivity were subsequently evaluated.

(E) Sequencing-based confirmation of the single residue mutation within *mepM*.

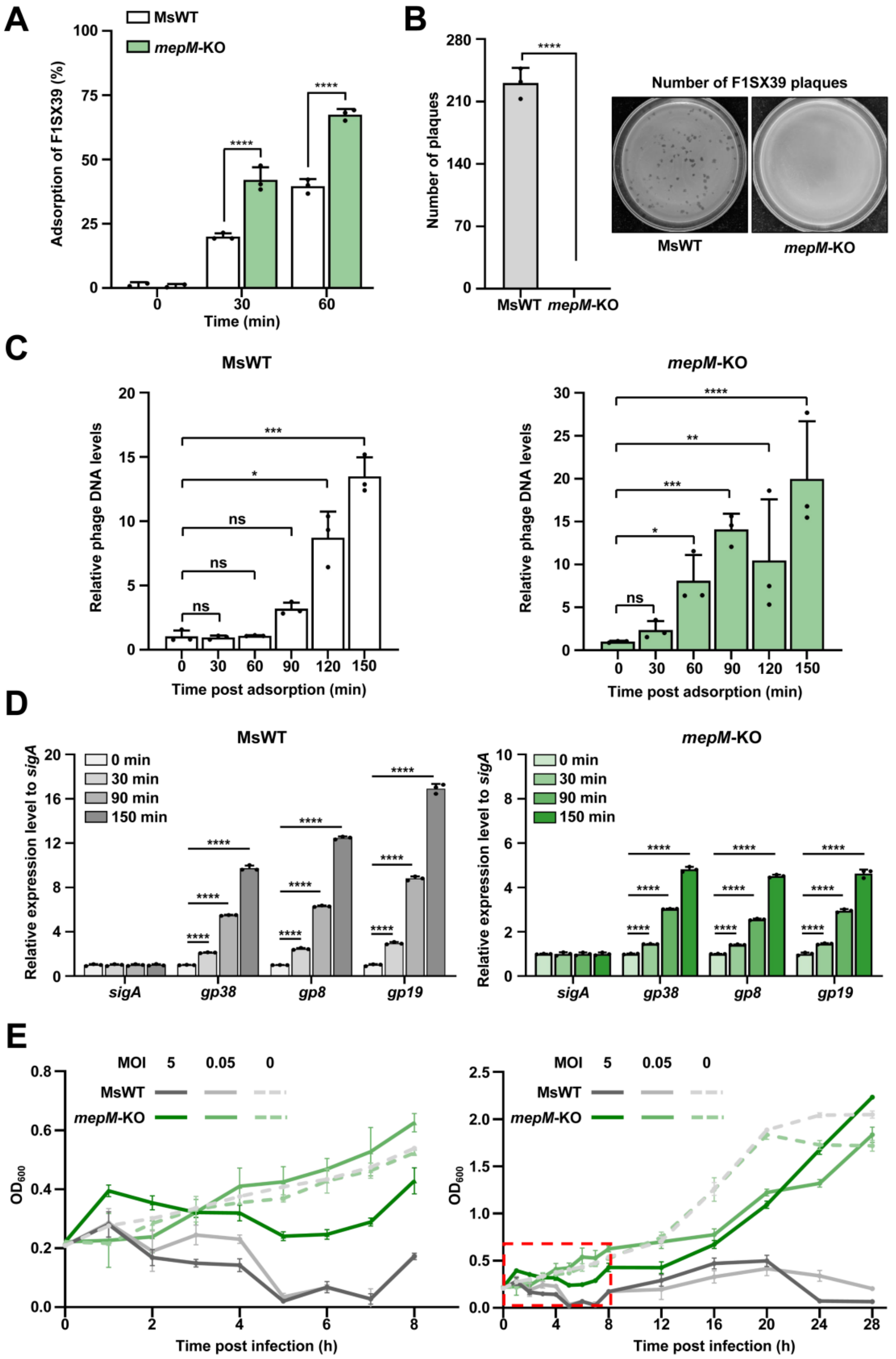

Fig. S3. *mepM* deletion does not affect the primary infection of F1SX39 but causes infection defects in its progeny phages

(A) Determination of the adsorption capacity of the F1SX39 phage on wild-type and *mepM*-KO strains.

(B) Determination of plaque formation ability after electroporation of F1SX39 phage

genomic DNA into wild-type and *mepM*-KO strains.
(C) Determination of phage DNA abundance in wild-type and *mepM*-KO strains following infection with F1SX39.
(D) Determination of phage gene expression in wild-type and *mepM*-KO strains following infection with F1SX39. *sigA* was used as a control.
(E) Growth curves for wild-type and *mepM*-KO strains under infection with the F1SX39 phage. All data in the quantitative analysis are expressed as mean ± standard deviation.
Biological replication: n = 3. Two-factor analysis of variance was performed using the GraphPad Prism software for *p*-value calculation. The asterisk indicates a significant difference between the two groups (ns indicates no significant difference, * indicates $p < 0.05$, ** indicates $p < 0.01$, *** indicates $p < 0.001$, *** indicates $p < 0.0001$).

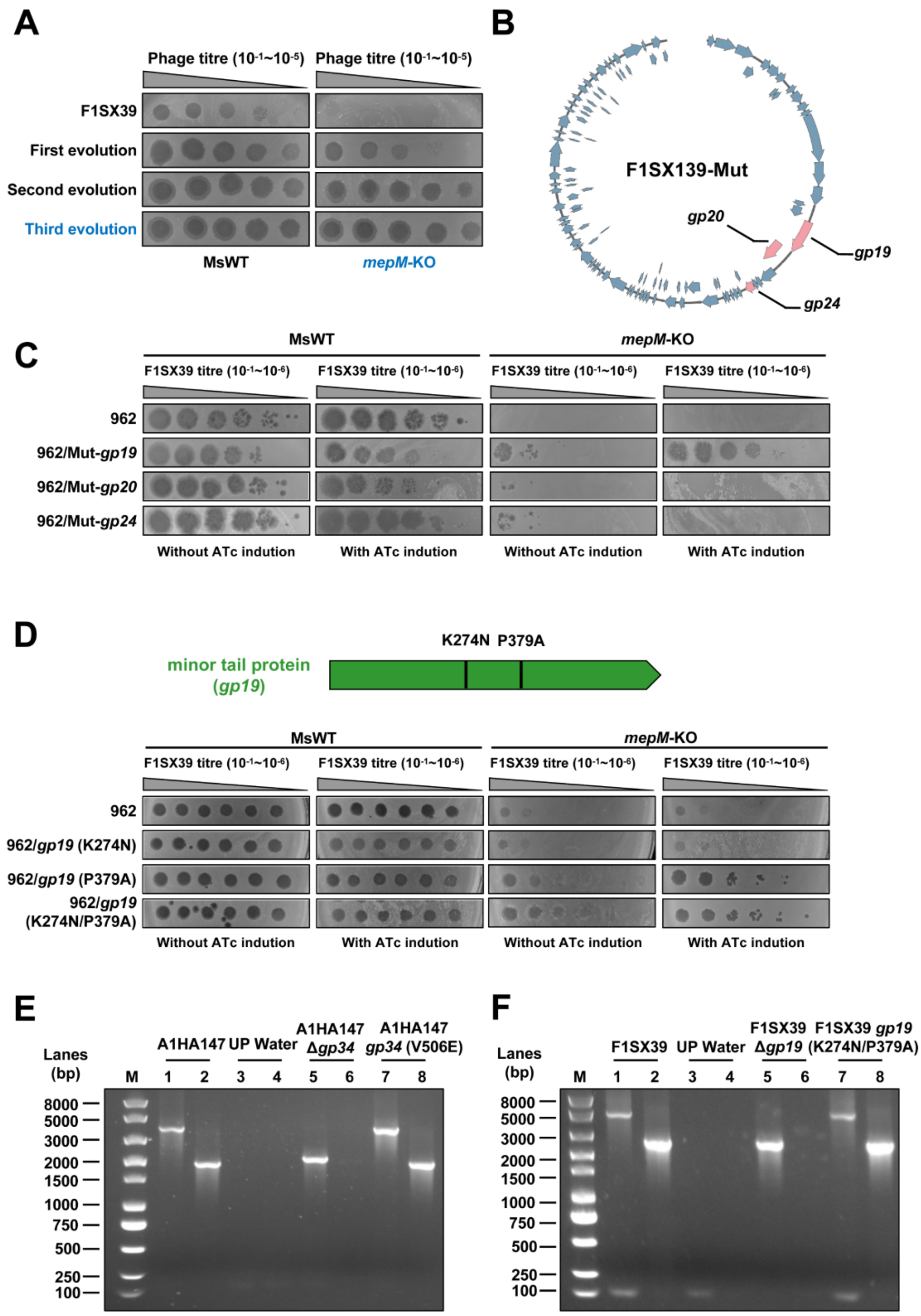

A
Phage titre (10-1~10-5)
Phage titre (10-1~10-5)
F1SX39
First evolution
Second evolution
Third evolution
MsWT
mepM-KO
B
F1SX139-Mut
gp20
gp19
gp24
C
MsWT
mepM-KO
F1SX39 titre (10-1~10-6)
962
962/Mut-gp19
962/Mut-gp20
962/Mut-gp24
Without ATc indution
With ATc indution
D
K274N P379A
minor tail protein (gp19)
MsWT
mepM-KO
F1SX39 titre (10-1~10-6)
962
962/gp19 (K274N)
962/gp19 (P379A)
962/gp19 (K274N/P379A)
Without ATc indution
With ATc indution
E
Lanes (bp)
M 1 2 3 4 5 6 7 8
A1HA147 UP Water A1HA147 Δgp34 A1HA147 gp34 (V506E)
8000 5000 3000 2000 1500 1000 750 500 250 100
F
Lanes (bp)
M 1 2 3 4 5 6 7 8
F1SX39 UP Water F1SX39 Δgp19 F1SX39 gp19 (K274N/P379A)
8000 5000 3000 2000 1500 1000 750 500 250 100

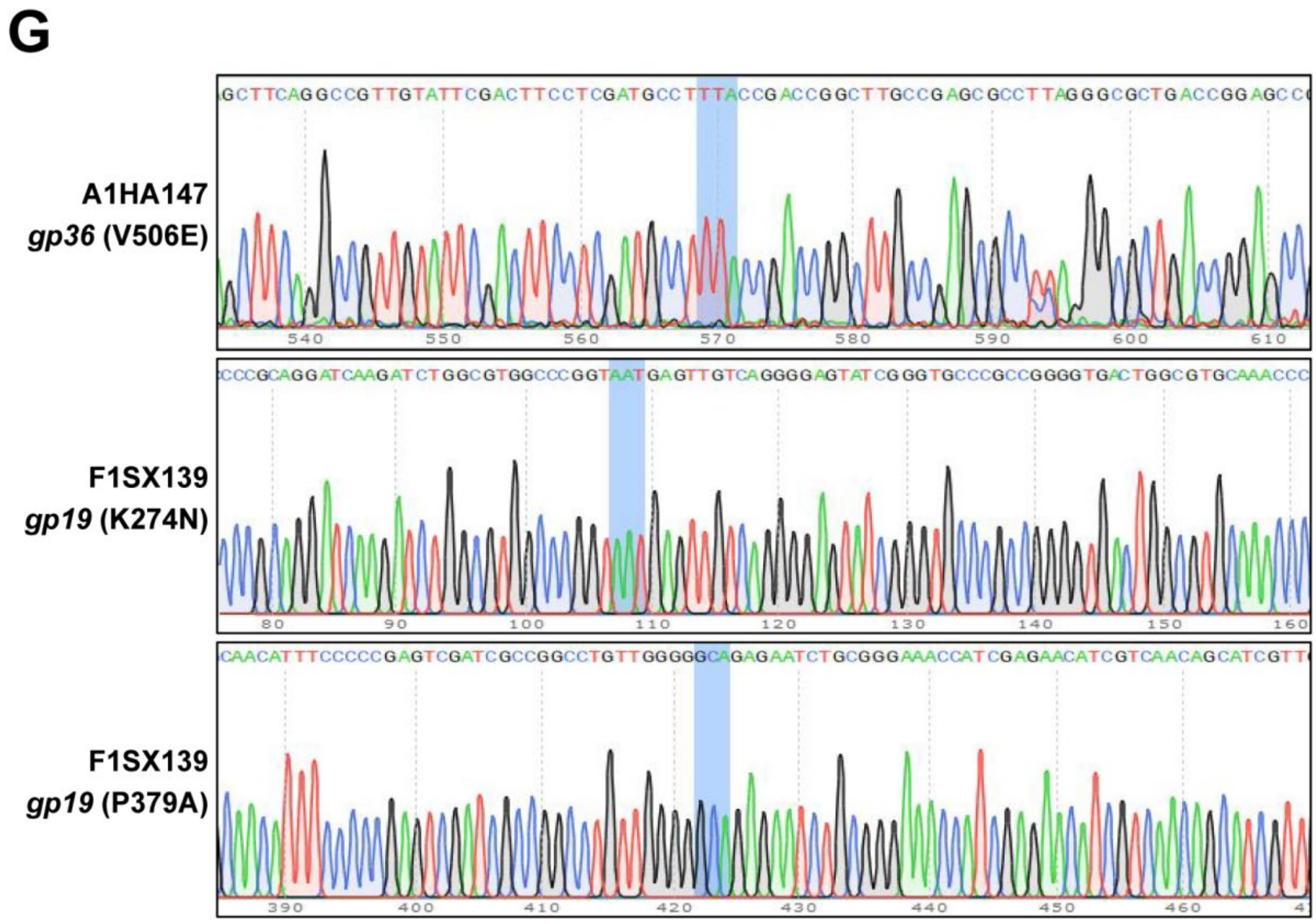

Fig. S4. Tail protein mutation restores phage infectivity of F1SX39 toward the *mepM*-KO strain.

(A) F1SX39 phage evolution causes restoration of infectivity toward the *mepM*-KO strain.

(B) Schematic showing the mutated gene in the evolutionary F1SX39-Mut. Three mutated genes within the genome of F1SX39-Mut, including *gp19*, *gp20*, and *gp24*, are marked in pink.

(C) Mut-*gp19* expression in the *mepM*-KO strain could partially restore F1SX39 phage infectivity, induced by 200 ng/mL ATc.

(D) F1SX39-Gp19 P379A mutant gene expression confers the F1SX39 phage with infectivity towards the *mepM*-KO strain.

(E) PCR validation of phage A1HA147, A1HA147-Δ *gp34*, and A1HA147-*gp34* (V506E), induced by 200 ng/mL ATc.

(F) PCR validation of phage F1SX39, F1SX39-Δ *gp19*, and F1SX39-*gp19* (K274N+P379A).

(G) Sequencing confirmation of wild-type *gp34* gene replaced by mutant *gp34* (V506E) in the genome of A1HA147-*gp34* (V506E) and confirmation of wild-type *gp19* gene replaced by mutant *gp19* (K274N+P379A) in the genome of F1SX39-*gp19* (K274N+P379A).

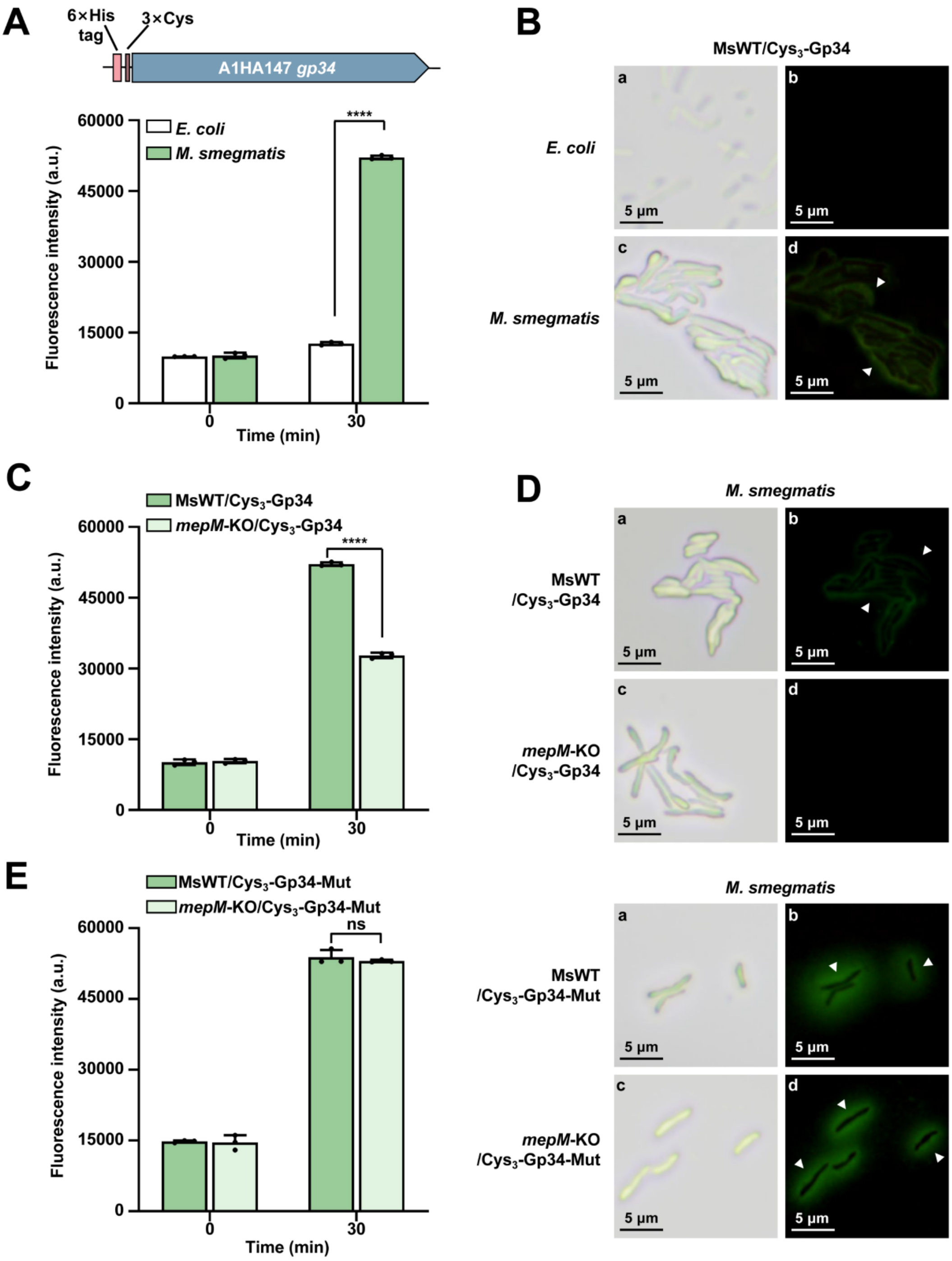

Fig. S5. Fluorescein-labelled tail proteins expressed in the *mepM*-KO strain exhibit adsorption defects.

(A) Quantitative analysis of the association of Cys3-Gp34 with the *E. coli* and *M. smegmatis* cell surface. Histagged-Cys3-Gp34 was labeled with fluorescein 5-MF, incubated with *E. coli* and *M. smegmatis* for 45 min, and subjected to a relative

fluorescence intensity assay. All data in the quantitative analysis are expressed as mean ± standard deviation (n = 3, biological replicates). Two-factor analysis of variance was performed using GraphPad Prism for *p*-value calculation. The asterisk indicates a significant difference between the two groups (ns indicates no significant difference, **** indicates $p < 0.0001$).
(B) Fluorescence microscopy assay for the binding of Cys_3-Gp34 to *E. coli* and *M. smegmatis*. Scale bar: 5 µm.
(C) Quantitative analysis of two different Cys_3-Gp34 proteins associated with the *M. smegmatis* cell surface. Two distinct fusion proteins, WT-Cys_3-Gp34 and KO-Cys_3-Gp34, expressed in wild-type and *mepM*-knockout *M. smegmatis*, respectively, were incubated with *M. smegmatis* for 45 min and subjected to a relative fluorescence intensity assay as described above. Two-factor analysis of variance was performed using GraphPad Prism for *p*-value calculation. The asterisk indicates a significant difference between the two groups (ns indicates no significant difference, **** indicates $p < 0.0001$). Biological replication: n = 3.
(D) Fluorescence microscopy assays for the binding of WT- Cys_3-Gp34 and KO- Cys_3-Gp34 to *M. smegmatis*. Scale bar: 5 µm.
(E) Quantitative (left panel) and fluorescence microscopy (right panel) assays for the binding of Cys_3-MutGp34 to *M. smegmatis*. Scale bar: 5 µm. Two-factor analysis of variance was performed using GraphPad Prism for *p*-value calculation. The asterisk indicates a significant difference between the two groups (ns indicates no significant difference, **** indicates $p < 0.0001$). Biological replication: n = 3.

A

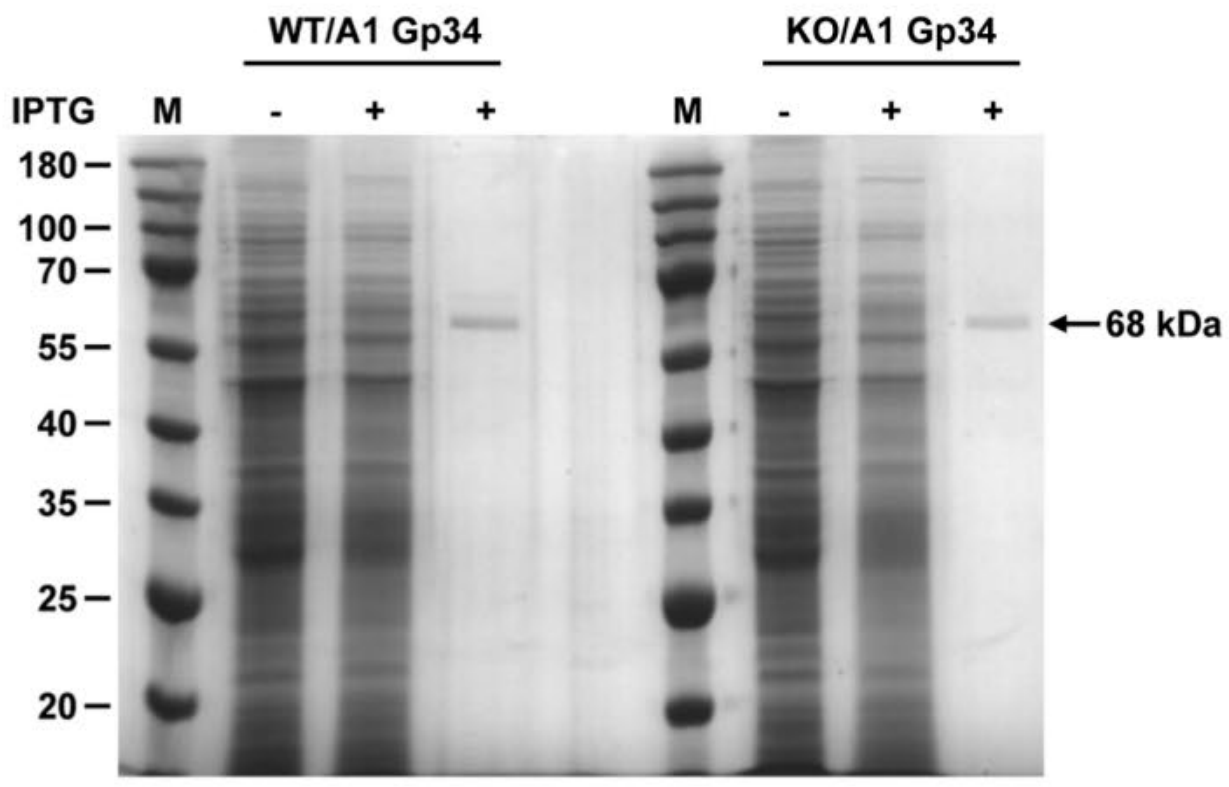

B

Sequence	Modifications
LAAGGDQHAYGGAGTKPGGGGNGGHWLGIYTQGGPGGPACAAVQFR	1xCarbamidomethyl [C40]
RIVDAIVSGIVGVTGIGAGISDLQSLIEQIASAAAR	
GEHFSGTTTILEWDGAELSIPGFEVSAANGSNGSGQRPVALGKPVGK	
VGIAVDTGTGSDHHDPQVEFFEKPVAIPVPAWCDRIDAIVTGK	1xCarbamidomethyl [C33]
VGIAVDTGTGSDHHDPQVEFFEKPVAIPVPAWCDRIDAIVTGK	1xCarbamidomethyl [C33]; 1xMethyl [D/E/I/K/R/S/T]
VGIAVDTGTGSDHHDPQVEFFEKPVAIPVPAWCDR	1xCarbamidomethyl [C33]
IVDAIVSGIVGVTGIGAGISDLQSLIEQIASAAAR	
IVDAIVSGIVGVTGIGAGISDLQSLIEQIASAAAR	1xMethyl [I/T]
KGALPGEVVGDGEGDVTPPNTSALHVDVSATTTSITITPSGAVDDA	
LAAGGDQHAYGGAGTKPGGGGNGGHWLGIYTQGGPGGPACAAVQFR	1xMethyl [H/I/K/L/N/Q/T]; 1xCarbamidomethyl [C40]
GGEGADGFLGFYGNPGLPGGVNTVTWTR	
VLEQILSYLPQELRDELEHAAAR	
IDAIVTGKGGEGADGFLGFYGNPGLPGGVNTVTWTR	
GGEGADGFLGFYGNPGLPGGVNTVTWTR	1xMethyl [D/E/L/N/T]
VGIAVDTGTGSDHHDPQVEFFEKPVAIPVPAWCDR	1xMethyl [D/E/H/K/S/T]; 1xCarbamidomethyl [C33]
SLSTPSLPPATLDKADVTWTDNVPR	1xMethyl [D/K/L/S/T]
SLSTPSLPPATLDKADVTWTDNVPR	
SLFQVPDFNPANALLVLPQLLLRLPLEALQK	
GEHFSGTTTILEWDGAELSIPGFEVSAANGSNGSGQRPVALGKPVGK	1xHexNAc [N/S/T]; 3xMethyl [D/E/N/Q/R/S/T]
DFIPNVLEGAFNTVAGAVDAIMGAIR	1xMethyl [D/I/N/T]
DFNLPDNDGAPIGNVGATR	
GGEGADGFLGFYGNPGLPGGVNTVTWTR	2xMethyl [E3; D6]
DFIPNVLEGAFNTVAGAVDAIMGAIR	1xOxidation [M22]
DFNLPDNDGAPIGNVGATR	1xMethyl [D/I/L/N/T]
VLEQILSYLPQELRDELEHAAAR	1xMethyl [D/E/H/L/R]
RIVDAIVSGIVGVTGIGAGISDLQSLIEQIASAAAR	2xMethyl [I]
DFIPNVLEGAFNTVAGAVDAIMGAIR	
SLFQVPDFNPANALLVLPQLLLR	
YGQDMTEDIAR	
GIEEVEYNGLK	
YGQDMTEDIAR	1xMethyl [D/E/I/Q/T]
SLSTPSLPPATLDKADVTWTDNVPR	2xMethyl [D/K/L/S/T]
LAAGGDQHAYGGAGTKPGGGGNGGHWLGIYTQGGPGGPACAAVQFRK	1xMethyl [D/L/Q]; 1xCarbamidomethyl [C40]
YGQDMTEDIAR	1xOxidation [M5]
DFNLPDNDGAPIGNVGATR	2xMethyl [D/I/L/N/R/T]
ADVTWTDNVPR	
GEHFSGTTTILEWDGAELSIPGFEVSAANGSNGSGQRPVALGKPVGK	1xMethyl [I/N/Q/R/S]
GRDFNLPDNDGAPIGNVGATR	
GEHFSGTTTILEWDGAELSIPGFEVSAANGSNGSGQRPVALGKPVGK	2xMethyl [E12; E17]; 1xHexNAc [N/S]
ADVTWTDNVPR	1xMethyl [D/N/T]
DELEHAAAR	
YGQDMTEDIAR	1xMethyl [D/E/Q/T]; 1xOxidation [M5]
SGLYKSER	
SLSTPSLPPATLDK	
LPLEALQK	

C

Sequence	Modifications
GEHFSGTTTILEWDGAELSIPGFEVSAANGSNGSGQRPVALGKPVGK	
VGIAVDTGTGSDHHDPQVEFFEKPVAIPVPAWCDRIDAIVTGK	1xCarbamidomethyl [C33]; 1xMethyl [D34]
VGIAVDTGTGSDHHDPQVEFFEKPVAIPVPAWCDRIDAIVTGK	1xCarbamidomethyl [C33]
IVDAIVSGIVGVTGIGAGISDLQSLIEQIASAAAR	
KGALPGEVVGDGEGDVTPPNTSALHVDVSATTTSITITPSGAVDDA	
IVDAIVSGIVGVTGIGAGISDLQSLIEQIASAAAR	1xMethyl [D/I/S/T]
DFIPNVLEGAFNTVAGAVDAIMGAIR	1xOxidation [M22]
GGEGADGFLGFYGNPGLPGGVNTVTWTR	
VLEQILSYLPQELRDELEHAAAR	
VGIAVDTGTGSDHHDPQVEFFEKPVAIPVPAWCDR	1xCarbamidomethyl [C33]
SLSTPSLPPATLDKADVTWTDNVPR	1xMethyl [D/K/L/N/R/S/T]
SLSTPSLPPATLDKADVTWTDNVPR	
RIVDAIVSGIVGVTGIGAGISDLQSLIEQIASAAAR	
SLFQVPDFNPANALLVLPQLLLR	
GGEGADGFLGFYGNPGLPGGVNTVTWTR	1xMethyl [D/E/L/N]
DFNLPDNDGAPIGNVGATR	
VLEQILSYLPQELRDELEHAAAR	1xMethyl [D/E/H/I/L/Q/R/S]
SLFQVPDFNPANALLVLPQLLLRLPLEALQK	
YGQDMTEDIAR	
DFIPNVLEGAFNTVAGAVDAIMGAIR	
YGQDMTEDIAR	1xMethyl [D/Q]
GEHFSGTTTILEWDGAELSIPGFEVSAANGSNGSGQRPVALGKPVGK	1xMethyl [D/E/I/L/S]
VGIAVDTGTGSDHHDPQVEFFEKPVAIPVPAWCDR	1xMethyl [D/E/H/I/K/Q/S/T]; 1xCarbamidomethyl [C33]
ADVTWTDNVPR	
GIEEVEYNGLK	
YGQDMTEDIAR	1xOxidation [M5]
SLSTPSLPPATLDKADVTWTDNVPR	2xMethyl [D/K/L/S/T]
ADVTWTDNVPR	1xMethyl [D/N/T]
SLSTPSLPPATLDK	
LPLEALQK	
DFNLPDNDGAPIGNVGATR	1xMethyl [D1]
SGLYKSER	
YGQDMTEDIAR	1xMethyl [D4]; 1xOxidation [M5]

D

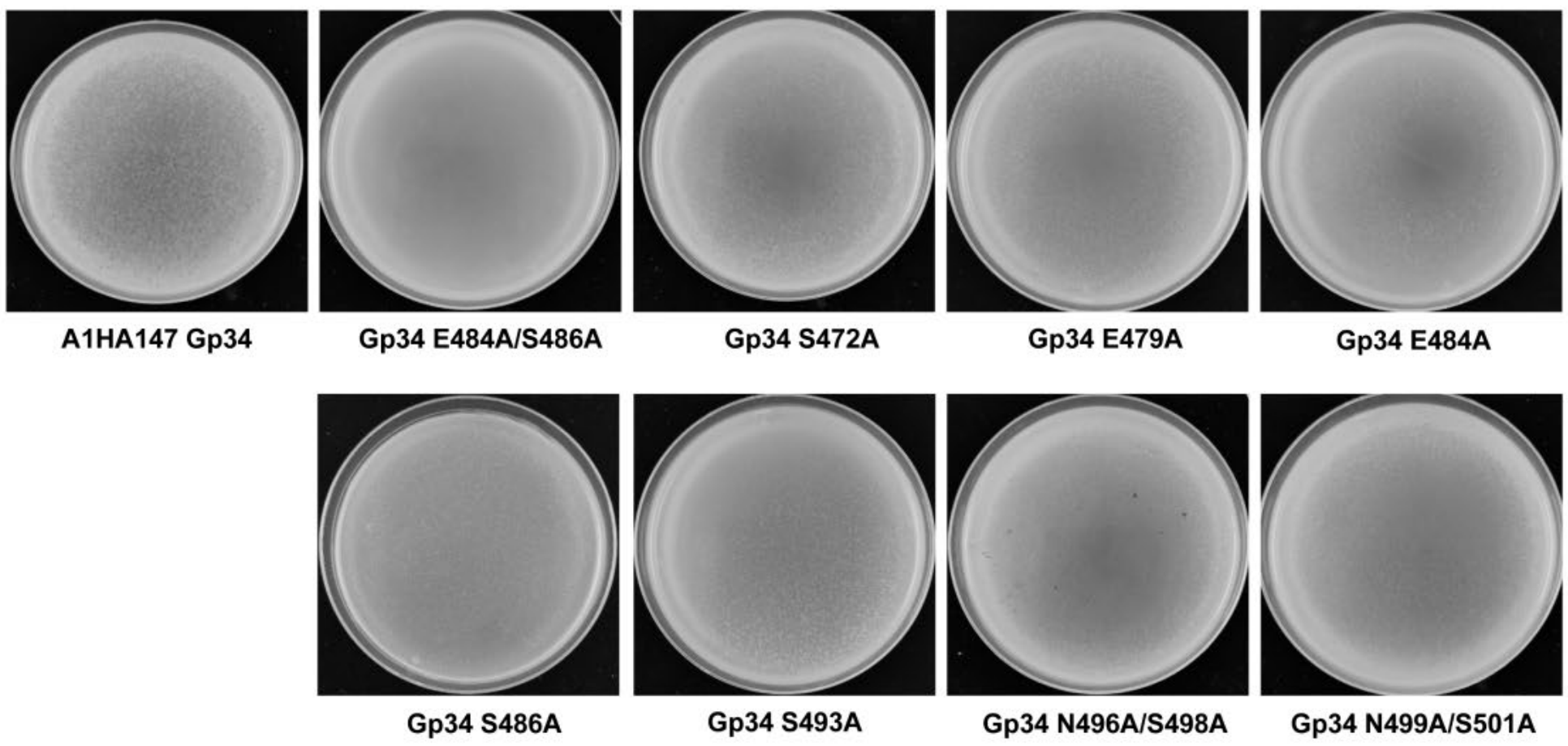

E

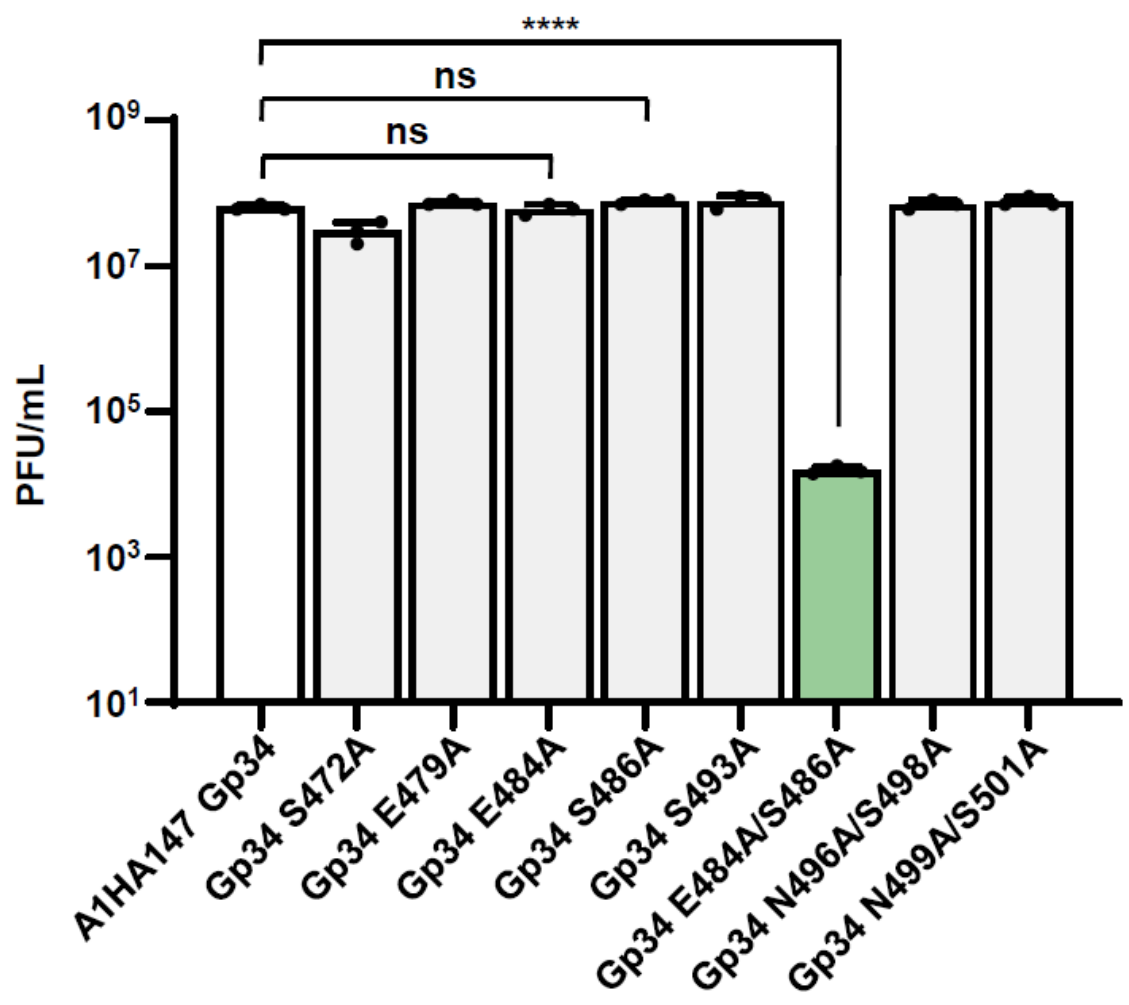

Fig. S6. Methylation-based modification of Gp34 is essential for progeny phage infectivity.

(A) SDS-PAGE-based analysis of A1HA147 Gp34 expressed in different strains. pMyNT-A1HA147 Gp34 expression vectors were transformed into wild-type and *mepM*-KO strains. Protein expression was induced through the addition of 0.2% acetamide (M/V) until the logarithmic growth phase. The purified proteins were treated with trypsin to degrade them into peptides of varying sizes, and then the peptides were subjected to mass spectrometric analysis.

(B) Analysis of WT-Gp34 methylation sites through liquid chromatography-mass spectrometry.

(C) Analysis of KO-Gp34 methylation sites through liquid chromatography-mass spectrometry.

(D) Effects of A1HA147-Gp34 modification site mutations on phage plaque formation efficiency. Point mutations were induced on PTM-prone amino acids that may exist in Gp34, and the mutated gene was transformed into the wild-type lysogenic bacteria, A1HA147 Δ *gp34*. Double-layered plates containing recombinant strains expressing these mutant Gp34 proteins were prepared for the determination of plaque formation efficiency. Following induction, the transformed bacteria were co-cultured with the Gp34-expressing recombinant strain on double-layered plates. Then, the effect of Gp34 mutations on the second-round phage infection was observed and assessed.

(E) Quantitative analysis of the second-round infection efficiency of the A1HA147-Gp34 phage and its mutants. All data are presented as mean ± standard deviation (n = 3, biological replicates). Two-factor analysis of variance was performed using GraphPad Prism for *p*-value calculation. The asterisk indicates a significant difference between two groups (ns indicates no significant difference).

Table S1 Bacterial strains used in this study

Strain name	Relevant genotype or feature	Source
E. coli DH5α		TaKaRa
M. smegmatis MC^2 155		ATCC
MsWT	*M. smegmatis* MC^2 155	ATCC
MutRS	*M. smegmatis* MC^2 155 with *MSMEG_6482* mutation	This study
mepM-KO	*M. smegmatis* MC^2 155 with *MSMEG_6482* deletion	This study
WT/962	*M. smegmatis* MC^2 155 with pLJR962	This study
KO/962	*mepM*-KO with pLJR962	This study
KO/Comp-*Ms6482*	*mepM*-KO with pLJR962-Comp-*Ms6482*	This study
KO/*Ms6482*-N176	*mepM*-KO with pLJR962-*Ms6482*-N176	This study
KO/*Ms6482*-Met	*mepM*-KO with pLJR962-*Ms6482*-Met	This study
KO/*Ms6482*-C175	*mepM*-KO with pLJR962-*Ms6482*-C175	This study
KO/Comp-*Ms6482* (E82A)	*mepM*-KO with pLJR962-Comp-*Ms6482* (E82A)	This study
KO/Comp-*Ms6482* (E83A)	*mepM*-KO with pLJR962-Comp-*Ms6482* (E83A)	This study
KO/Comp-*Ms6482* (D146A)	*mepM*-KO with pLJR962-Comp-*Ms6482* (D146A)	This study
KO/Comp-*Ms6482* (H149A)	*mepM*-KO with pLJR962-Comp-*Ms6482* (H149A)	This study
KO/Comp-*Ms6482* (D175A)	*mepM*-KO with pLJR962-Comp-*Ms6482* (D175A)	This study
KO/Comp-*Ms6482* (F177A)	*mepM*-KO with pLJR962-Comp-*Ms6482* (F177A)	This study
WT 962/Mut-*gp34*	*M. smegmatis* MC^2 155 with pLJR962-Mut-*gp34*	This study
WT 962/Mut-*gp25*	*M. smegmatis* MC^2 155 with pLJR962-Mut-*gp25*	This study
WT 962/Mut-*gp74*	*M. smegmatis* MC^2 155 with pLJR962-Mut-*gp74*	This study
WT 962/Mut-*gp67*	*M. smegmatis* MC^2 155 with pLJR962-Mut-*gp67*	This study
KO 962/Mut-*gp34*	*mepM*-KO with pLJR962-Mut-*gp34*	This study
KO 962/Mut-*gp25*	*mepM*-KO with pLJR962-Mut-*gp25*	This study
KO 962/Mut-*gp74*	*mepM*-KO with pLJR962-Mut-*gp74*	This study
KO 962/Mut-*gp67*	*mepM*-KO with pLJR962-Mut-*gp67*	This study
WT 962/*gp34* (K171L)	*M. smegmatis* MC^2 155 with pLJR962-*gp34* (K171L)	This study
WT 962/*gp34* (D445H)	*M. smegmatis* MC^2 155 with pLJR962-*gp34* (D445H)	This study
WT 962/*gp34* (A507V)	*M. smegmatis* MC^2 155 with pLJR962-*gp34* (A507V)	This study
WT 962/*gp34* (V506E)	*M. smegmatis* MC^2 155 with pLJR962-*gp34* (V506E)	This study
KO 962/*gp34* (K171L)	*mepM*-KO with pLJR962-*gp34* (K171L)	This study
KO 962/*gp34* (D445H)	*mepM*-KO with pLJR962-*gp34* (D445H)	This study
KO 962/*gp34* (A507V)	*mepM*-KO with pLJR962-*gp34* (A507V)	This study
KO 962/*gp34* (V506E)	*mepM*-KO with pLJR962-*gp34* (V506E)	This study
WT 962/Mut-*gp24*	*M. smegmatis* MC^2 155 with pLJR962-Mut-*gp24*	This study

Strain name	Relevant genotype or feature	Source
WT 962/Mut-*gp20*	*M. smegmatis* MC^2 155 with pLJR962-Mut-*gp20*	This study
WT 962/Mut-*gp19*	*M. smegmatis* MC^2 155 with pLJR962-Mut-*gp19*	This study
KO 962/Mut-*gp24*	*mepM*-KO with pLJR962-Mut-*gp24*	This study
KO 962/Mut-*gp20*	*mepM*-KO with pLJR962-Mut-*gp20*	This study
KO 962/Mut-*gp19*	*mepM*-KO with pLJR962-Mut-*gp19*	This study
WT 962/*gp19* (K274N)	*M. smegmatis* MC^2 155 with pLJR962-*gp19* (K274N)	This study
WT 962/*gp19* (P379A)	*M. smegmatis* MC^2 155 with pLJR962-*gp19* (P379A)	This study
WT 962/*gp19* (K274N/P379A)	*M. smegmatis* MC^2 155 with pLJR962-*gp19* (K274N/P379A)	This study
KO 962/*gp19* (K274N)	*mepM*-KO with pLJR962-*gp19* (K274N)	This study
KO 962/*gp19* (P379A)	*mepM*-KO with pLJR962-*gp19* (P379A)	This study
KO 962/*gp19* (K274N/P379A)	*mepM*-KO with pLJR962-*gp19* (K274N/P379A)	This study
Msm (A1)	*M. smegmatis* MC^2 155 with prophage A1HA147	This study
Msm (A1-Δ*gp34*)	*M. smegmatis* MC^2 155 with prophage A1HA147-Δ*gp34*	This study
Msm (F1)	*M. smegmatis* MC^2 155 with prophage F1SX39	This study
Msm (F1-Δ*gp19*)	*M. smegmatis* MC^2 155 with prophage F1SX39-Δ*gp19*	This study
WT/pMyNT-EGFP-Gp34	*M. smegmatis* MC^2 155 with pMyNT-EGFP-Gp34	This study
KO/pMyNT-EGFP-Gp34	*mepM*-KO with pMyNT-EGFP-Gp34	This study
WT/pMyNT-Cys_3-Gp34	*M. smegmatis* MC^2 155 with pMyNT-Cys_3-Gp34	This study
KO/pMyNT-Cys_3-Gp34	*mepM*-KO with pMyNT-Cys_3-Gp34	This study
WT/pMyNT-EGFP-Mut-Gp34	*M. smegmatis* MC^2 155 with pMyNT-EGFP-Mut-Gp34	This study
KO/pMyNT-EGFP-Mut-Gp34	*mepM*-KO with pMyNT-EGFP-Mut-Gp34	This study
WT/pMyNT-Cys_3-Mut-Gp34	*M. smegmatis* MC^2 155 with pMyNT-Cys_3-Mut-Gp34	This study
KO/pMyNT-Cys_3-Mut-Gp34	*mepM*-KO with pMyNT-Cys_3-Mut-Gp34	This study
Msm (A1-Δ*gp34*)/Gp34	Msm (A1-Δ*gp6*) with pLJR962-*gp34*	This study
Msm (A1-Δ*gp34*)/Gp34 (S472A)	Msm (A1-Δ*gp6*) with pLJR962-*gp34* (S472A)	This study
Msm (A1-Δ*gp34*)/Gp34 (E479A)	Msm (A1-Δ*gp6*) with pLJR962-*gp34* (E479A)	This study
Msm (A1-Δ*gp34*)/Gp34 (E484A)	Msm (A1-Δ*gp6*) with pLJR962-*gp34* (E484A)	This study
Msm (A1-Δ*gp34*)/Gp34 (S486A)	Msm (A1-Δ*gp6*) with pLJR962-*gp34* (S486A)	This study
Msm (A1-Δ*gp34*)/Gp34 (S493A)	Msm (A1-Δ*gp6*) with pLJR962-*gp34* (S493A)	This study
Msm (A1-Δ*gp34*)/Gp34 (E484A/S486A)	Msm (A1-Δ*gp6*) with pLJR962-*gp34* (E484A/S486A)	This study
Msm (A1-Δ*gp34*)/Gp34 (N496A/S498A)	Msm (A1-Δ*gp6*) with pLJR962-*gp34* (N496A/S498A)	This study
Msm (A1-Δ*gp34*)/Gp34 (N499A/S501A)	Msm (A1-Δ*gp6*) with pLJR962-*gp34* (N499A/S501A)	This study

Table S2 Primers used in this study

Primer name	Primer sequence (5' to 3')	Usage
Ms6482 up1000-F	ATATATTTAATTAATCGAGCACATCCGCTCGGCCGTCA	Knockout
Ms6482 up1000-R	GTGCGTGGTCACCGACCGCTAGGGGTGACAACATACCGTTGCTGG	Knockout
Ms6482 dn1000-F	ACCAGCAACGGTATGTTGTCACCCCTAGCGGTCGGTGACCACGCA	Knockout
Ms6482 dn1000-R	ATATAAAAGCTAGCCATCGCGGCCACCCAGGAGTCCT	Knockout
A1 *gp34* up1000-F	ATATATTTAATTAATGTGCATGTGGACTCTCTGCGCCGGCTCGCTGACGAGA	Knockout
A1 *gp34* dn1000-R	ATATAAAAGCTAGCGCGCGGTAACCCGCACCGCCGCCACCGCCGCCGCC	Knockout
A1 *gp19* up1250-F	ATATATTTAATTAATGAGCGGTTCTATCCGACAGCCTCGG	Knockout
A1 *gp19* dn1254-R	ATATAAAAGCTAGCCGTTCCGGGATACAGATACTCCGCCACC	Knockout
sigA-F	CGAGGAAGAAGAAGCTGATG	qPCR
sigA-R	CGTCTTTGCGTGCCTGTC	qPCR
A1 *gp46*-F	GAAGGTCGAGGACACCAAGA	qPCR
A1 *gp46*-R	CACCGGGTCGATGTAGAACT	qPCR
A1 *gp34*-F	AAGACTTCATCCCGAACGTG	qPCR
A1 *gp34*-R	AGGTCTTCGATCGTGTGTCC	qPCR
A1 *gp25*-F	CGAGCTGCGTAAGGATGTCT	qPCR
A1 *gp25*-R	TGAGCACGATTCTCAACCTG	qPCR
F1 *gp38*-F	TAACCTCGGGCTGGTCATAC	qPCR
F1 *gp38*-R	CCGGAATCACCGAACAATAC	qPCR
F1 *gp8*-F	GCAACCACATCGAACACATC	qPCR
F1 *gp8*-R	TACACGGTGACGCACTATCC	qPCR
F1 *gp19*-F	TCGAGAACATCGTCAACAGC	qPCR
F1 *gp19*-R	AAGGCAACAACCCACTATCG	qPCR
Cys_3-Gp34-F	GGACGATAAGGTACCGGATCCGTGCTGCTGCGTGGCTGACGACCAGTG	Protein expression
Cys_3-Gp34-R	CGTCGACATCGATAAGCTTGCTAGGCATCGTCGACAGCTCCC	Protein expression
EGFP-F	ATGGACGATAAGGTACCGGATCCGATGGTGAGCAAGGGCGA	Protein expression
EGFP-R	GGTCGTCAGCCACCGATCCACCTCCACCCGATCCACCTCCACCCTTGTACAGCTCG	Protein expression
EGFP-Gp34-F	GGACGAGCTGTACAAGGGTGGAGGTGGATCGGGTGGAGGTGGATCGGTGGCTG	Protein expression
EGFP-Gp34-R	AGTTAACTACGTCGACATCGATAAGCTTCTAGGCATCGTCGACAGCTCCCG	Protein expression
Gp34-F	GACGATAAGGTACCGGATCCGGTGGCTGACGACCAGTGGGT	Protein expression
Gp34-R	TACGTCGACATCGATAAGCTTCTAGGCATCGTCGACAGCTCCCGA	Protein expression
Ms6482-F	TGATAGAGAAGGCGGTATCGATATGAGCCATGCCGACACC	Inducible expression
Ms6482-R	AAAGAAAAATGGCCAGAGTATCTAGTCGAGCGTGCCGG	Inducible expression

Primer name	Primer sequence (5' to 3')	Usage
Ms6482 (1-176)-F	AGTGATAGAGAAGGCGGTATCGATATGAGCCATGCCGACACCGCA	Inducible expression
Ms6482 (1-176)-R	TGCTTAGCTAATCAGCGGCCGCCTACACGTCGTGGATGACCAG	Inducible expression
Ms6482 (40-180)-F	AGTGATAGAGAAGGCGGTATCGATATGCGCTACCTCGGCGGCGGCGT	Inducible expression
Ms6482 (40-180)-R	TCTGCTTAGCTAATCAGCGGCCGCCTAGGGATTCGGGAACACGT	Inducible expression
Ms6482 (48-223)-F	AGTGATAGAGAAGGCGGTATCGATATGGTCGAGATCGGCACCTACT	Inducible expression
Ms6482 (48-223)-R	TGCTTAGCTAATCAGCGGCCGCCTAGTCGAGCGTGCCGGCCTCT	Inducible expression
Ms6482 (E82A)-F	CACCACCACGGCTCCGCAGAGCACCAGCCCGG	Inducible expression
Ms6482 (E82A)-R	CCGGGCTGGTGCTCTGCGGAGCCGTGGTGGTG	Inducible expression
Ms6482 (E83A)-F	ACCACCACCACGGCTCCGAGGCGCACCAGCCCGGCTGGGAGTA	Inducible expression
Ms6482 (E83A)-R	TACTCCCAGCCGGGCTGGTGCGCCTCGGAGCCGTGGTGGTGGT	Inducible expression
Ms6482 (D146A)-F	GTTGCGGTTCCTGTTCATCGCCGGCGGTCACACCGAGGAGGC	Inducible expression
Ms6482 (D146A)-R	GCCTCCTCGGTGTGACCGCCGGCGATGAACAGGAACCGCAAC	Inducible expression
Ms6482 (H149)-F	TCCTGTTCATCGACGGCGGTGCCACCGAGGAGGCCGCGCAGCGCGA	Inducible expression
Ms6482 (H149A)-R	TCGCGCTGCGCGGCCTCCTCGGTGGCACCGCCGTCGATGAACAGGA	Inducible expression
Ms6482 (D175A)-F	CGGCGCCCTGGTCATCCACGCCGTGTTCCCGAATCCCGACGA	Inducible expression
Ms6482 (D175A)-R	TCGTCGGGATTCGGGAACACGGCGTGGATGACCAGGGCGCCG	Inducible expression
Ms6482 (F177A)-F	CGCCCTGGTCGCACACGACGTGTTCCCGAATC	Inducible expression
Ms6482 (F177A)-R	GATTCGGGAACACGTCGTGTGCGACCAGGGCG	Inducible expression
A1 *gp34*-F	GATAGAGAAGGCGGTATCGATGTGGCTGACGACCAGTGGGT	Inducible expression
A1 *gp34*-R	TAGCTAATCAGCGGCCGCCTAGGCATCGTCGACAGCT	Inducible expression
A1 *gp25*-F	ATAGAGAAGGCGGTATCGATATGAGCACGATTCTCAACCTGG	Inducible expression
A1 *gp25*-R	AGCTAATCAGCGGCCGCTCAGTCCTCCGAGCTGGAGGCT	Inducible expression
A1 *gp74*-F	ATAGAGAAGGCGGTATCGATATGAGAACCACCAGAGAACAG	Inducible expression
A1 *gp74*-R	TGCTTAGCTAATCAGCGGCCGCTCAGGGCTCCACGCTCGGG	Inducible expression
A1 *gp67*-F	ATAGAGAAGGCGGTATCGATGTGACCGAACGATGGA	Inducible expression
A1 *gp67*-R	TTAGCTAATCAGCGGCCGCTCACTTCGCACCTCTC	Inducible expression
A1 *gp34* (K171L)-F	CGGGTCGATCGAAGAGACCATCCTGCGCATCGTCGATGCGATCGTCTCGG	Inducible expression
A1 *gp34* (K171L)-R	CCGAGACGATCGCATCGACGATGCGCAGGATGGTCTCTTCGATCGACCCG	Inducible expression
A1 *gp34* (D445H)-F	CACCGGTAAGGGCGGCGAGGGTGCCCACGGGTTCCTCGGGTTCTACGGCAAC	Inducible expression
A1 *gp34* (D445H)-R	GTTGCCGTAGAACCCGAGGAACCCGTGGGCACCCTCGCCGCCCTTACCGGTG	Inducible expression
A1 *gp34* (A507V)-F	CGGCTCCGGTCAGCGCCCTGTGGTGCTCGGCAAGCCGGTCGGTAAAGG	Inducible expression
A1 *gp34* (A507V)-R	CCTTTACCGACCGGCTTGCCGAGCACCACAGGGCGCTGACCGGAGCCG	Inducible expression
A1 *gp34* (V506E)-F	CTAACGGCTCCGGTCAGCGCCCTGAGGCGCTCGGCAAGCCGGTCGGTAAAG	Inducible expression

Primer name	Primer sequence (5' to 3')	Usage
A1 *gp34* (V506E)-R	CTTTACCGACCGGCTTGCCGAGCGCCTCAGGGCGCTGACCGGAGCCGTTAG	Inducible expression
F1 *gp24*-F	ATAGAGAAGGCGGTATCGATATGGCTTATTCGAAGCA	Inducible expression
F1 *gp24*-R	AAACGATGACGTCATTCACGGCACCACCAC	Inducible expression
F1 *gp20*-F	AGAGAAGGCGGTATCGATATGACAATCAAAGGGT	Inducible expression
F1 *gp20*-R	TGCTTAGCTAATCAGCGGCCGCTCATCCCCCAATCTGCAAGTTCAC	Inducible expression
F1 *gp19*-F	ATAGAGAAGGCGGTATCGATATGACGTCATCGTTTGATCCGTTG	Inducible expression
F1 *gp19*-R	GCTTAGCTAATCAGCGGCCGCTCATACGTCTGTTGCTCCAGACCACG	Inducible expression
A1 *gp19* (K274N)-F	AGATCTGGCGTGGCCCGGTAATGAGTTGTCAGGGGAGTATCGG	Inducible expression
A1 *gp19* (K274N)-R	CCGATACTCCCCTGACAACTCATTACCGGGCCACGCCAGATCTTG	Inducible expression
A1 *gp19* (P379A)-F	CGATCGCCGGCCTGTTGGGGGCAGAGAATCTGCGGGAAACCATCG	Inducible expression
A1 *gp19* (P379A)-R	CGATGGTTTCCCGCAGATTCTCTGCCCCCAACAGGCCGGCGATCG	Inducible expression
A1 *gp34* (S472A)-F	TGAGCACTTCGCCGGTACCACCA	Inducible expression
A1 *gp34* (S472A)-R	TGGTGGTACCGGCGAAGTGCTCA	Inducible expression
A1 *gp34* (E479A)-F	CACGATCTTGGCGTGGGACGGCG	Inducible expression
A1 *gp34* (E479A)-R	CGCCGTCCCACGCCAAGATCGTG	Inducible expression
A1 *gp34* (E484A)-F	ACCCGGGGATCGACAGCGCAGCGCCGTCC	Inducible expression
A1 *gp34* (E484A)-R	GGACGGCGCTGCGCTGTCGATCCCCGGGT	Inducible expression
A1 *gp34* (S486A)-F	ACCCGGGGATCGCCAGCTCAGCGCCGTCC	Inducible expression
A1 *gp34* (S486A)-R	GGACGGCGCTGAGCTGGCGATCCCCGGGT	Inducible expression
A1 *gp34* (S493A)-F	GTTCGAGGTGGCCGCTGCCAACG	Inducible expression
gp34 (S493A)-R	CGTTGGCAGCGGCCACCTCGAAC	Inducible expression
gp34 (NGS-1)-F	CGGAGCCGTTTGCTCCTGCGGCAGCGGAC	Inducible expression
gp34 (NGS-1)-R	GTCCGCTGCCGCAGGAGCAAACGGCTCCG	Inducible expression
gp34 (ELS)-F	ACCCGGGGATCGCCAGCGCAGCGCCGTCC	Inducible expression
gp34 (ELS)-R	GGACGGCGCTGCGCTGGCGATCCCCGGGT	Inducible expression
gp34 (NGS-2)-F	GGCGCTGACCTGCGCCTGCAGATCCGTTG	Inducible expression
gp34 (NGS-2)-R	CAACGGATCTGCAGGCGCAGGTCAGCGCC	Inducible expression

Table S3 Plasmids used in this study

Plasmid name	Relevant genotype or feature	Source
pLJR962	Kan^r, Sth1 sgRNA scaffold and *dCas9*, TetR, L5 attP and Int for *M. smegmatis*	Addgene
pMind-*LacZ-SacB*	Kan^r, *lacZ* operator and promotor, *LacZ*, *SacB*	This study
pMyNT	Kan^r, 6×His, TEV site	Addgene
pMind-*Ms6482* UD	Flanking sequence of *Ms6482* inserted in *Pac*I-*Nhe*I of pMind-*LacZ-SacB*	This study
pMind-A1 *gp34* UD	Flanking sequence of *gp34* inserted in *Pac*I-*Nhe*I of pMind-*LacZ-SacB*	This study
pMind-F1 *gp19* UD	Flanking sequence of *gp19* inserted in *Pac*I-*Nhe*I of pMind-*LacZ-SacB*	This study
pMind-Mut-A1 *gp34* UD	Flanking sequence of Mut-*gp34* inserted in *Pac*I-*Nhe*I of pMind-*LacZ-SacB*	This study
pMind-Mut-F1 *gp19* UD	Flanking sequence of Mut-*gp19* inserted in *Pac*I-*Nhe*I of pMind-*LacZ-SacB*	This study
pLJR962-Comp-*Ms6482*	*Ms6482* inserted in *Cla*I-*Not*I of pLJR962	This study
pLJR962-*Ms6482*-N176	*Ms6482*-N176 inserted in *Cla*I-*Not*I of pLJR962	This study
pLJR962-*Ms6482*-Met	*Ms6482*-Met inserted in *Cla*I-*Not*I of pLJR962	This study
pLJR962-*Ms6482*-C175	*Ms6482*-C175 inserted in *Cla*I-*Not*I of pLJR962	This study
pLJR962-Comp-*Ms6482* (E82A)	*Ms6482* (E82A) inserted in *Cla*I-*Not*I of pLJR962	This study
pLJR962-Comp-*Ms6482* (E83A)	*Ms6482* (E83A) inserted in *Cla*I-*Not*I of pLJR962	This study
pLJR962-Comp-*Ms6482* (D146A)	*Ms6482* (D146A) inserted in *Cla*I-*Not*I of pLJR962	This study
pLJR962-Comp-*Ms6482* (H149A)	*Ms6482* (H149A) inserted in *Cla*I-*Not*I of pLJR962	This study
pLJR962-Comp-*Ms6482* (D175A)	*Ms6482* (D175A) inserted in *Cla*I-*Not*I of pLJR962	This study
pLJR962-Comp-*Ms6482* (F177A)	*Ms6482* (F177A) inserted in *Cla*I-*Not*I of pLJR962	This study
pLJR962-Mut-*gp34*	Mut-*gp34* inserted in *Cla*I-*Not*I of pLJR962	This study
pLJR962-Mut-*gp25*	Mut-*gp25* inserted in *Cla*I-*Not*I of pLJR962	This study
pLJR962-Mut-*gp74*	Mut-*gp74* inserted in *Cla*I-*Not*I of pLJR962	This study
pLJR962-Mut-*gp67*	Mut-*gp67* inserted in *Cla*I-*Not*I of pLJR962	This study
pLJR962-*gp34* (K171L)	*gp34* (K171L) inserted in *Cla*I-*Not*I of pLJR962	This study
pLJR962-*gp34* (D445H)	*gp34* (D445H) inserted in *Cla*I-*Not*I of pLJR962	This study
pLJR962-*gp34* (A507V)	*gp34* (A507V) inserted in *Cla*I-*Not*I of pLJR962	This study
pLJR962-*gp34* (V506E)	*gp34* (V506E) inserted in *Cla*I-*Not*I of pLJR962	This study
pLJR962-Mut-*gp24*	Mut-*gp24* inserted in *Cla*I-*Not*I of pLJR962	This study
pLJR962-Mut-*gp20*	Mut-*gp20* inserted in *Cla*I-*Not*I of pLJR962	This study
pLJR962-Mut-*gp19*	Mut-*gp19* inserted in *Cla*I-*Not*I of pLJR962	This study
pLJR962-*gp19* (K274N)	*gp19* (K274N) inserted in *Cla*I-*Not*I of pLJR962	This study
pLJR962-*gp19* (P379A)	*gp19* (P379A) inserted in *Cla*I-*Not*I of pLJR962	This study

Plasmid name	Relevant genotype or feature	Source
pLJR962-*gp19* (K274N/P379A)	*gp19* (K274N/P379A) inserted in *Cla*I-*Not*I of pLJR962	This study
pMyNT-EGFP-Gp34	*egfp*-(GGGS)$_2$-*gp34* inserted in *Bam*HI-*Hind*Ⅲ of pMyNT	This study
pMyNT-Cys_3-Gp34	Cys_3-*gp34* inserted in *Bam*HI-*Hind*Ⅲ of pMyNT	This study
pMyNT-EGFP-Mut-Gp34	*egfp*-(GGGS)$_2$-Mut-*gp34* inserted in *Bam*HI-*Hind*Ⅲ of pMyNT	This study
pMyNT-Cys_3-Mut-Gp34	Cys_3-Mut-*gp34* inserted in *Bam*HI-*Hind*Ⅲ of pMyNT	This study
pLJR962-*gp34*	*gp34* inserted in *Cla*I-*Not*I of pLJR962	This study
pLJR962-*gp34* (S472A)	*gp34* (S472A) inserted in *Cla*I-*Not*I of pLJR962	This study
pLJR962-*gp34* (E479A)	*gp34* (E479A) inserted in *Cla*I-*Not*I of pLJR962	This study
pLJR962-*gp34* ((E484A)	*gp34* (E484A) inserted in *Cla*I-*Not*I of pLJR962	This study
pLJR962-*gp34* (S486A)	*gp34* (E486A) inserted in *Cla*I-*Not*I of pLJR962	This study
pLJR962-*gp34* (S493A)	*gp34* (E493A) inserted in *Cla*I-*Not*I of pLJR962	This study
pLJR962-*gp34* (E484A/S486A)	*gp34* (E484A/S486A) inserted in *Cla*I-*Not*I of pLJR962	This study
pLJR962-*gp34* (N496A/S498A)	*gp34* (N496A/S498A) inserted in *Cla*I-*Not*I of pLJR962	This study
pLJR962-*gp34* (N499A/S501A)	*gp34* (N499A/S501A) inserted in *Cla*I-*Not*I of pLJR962	This study
